# Integrated multidisciplinary approach for reducing uncertainty in reservoir characterization of the Bahariya Formation, Berenice Field, Egypt

**DOI:** 10.1038/s41598-026-47694-1

**Published:** 2026-04-30

**Authors:** Michael Nabil Fawzy, Tamer Mohamed Salem, Abdel Nasser Helal, Amir Maher Sayed Lala

**Affiliations:** Department of Geophysics, Ain Shams University, Cairo, Egypt

**Keywords:** Western desert, Reservoir characterization, Seismic attributes, Facies modeling, Uncertainty analysis, Petrophysical evaluation, Hydrocarbon detection, Structural trapping, Berenice oil field, Energy science and technology, Engineering, Solid Earth sciences

## Abstract

This study presents an integrated static reservoir characterization of the Upper Cretaceous Bahariya Formation in the Berenice Field, North Western Desert, Egypt. A multidisciplinary workflow combining geological, geophysical, and petrophysical techniques was applied to minimize subsurface uncertainty and enhance hydrocarbon prediction. Seismic interpretation established the structural framework and fault geometries controlling reservoir distribution. A synthetic seismogram was generated to achieve precise well-to-seismic ties, improving the correlation between log-derived parameters and seismic reflectors. Seismic attributes, including variance, dip angle, and dip azimuth, were analyzed to delineate subtle structural and stratigraphic features that are not apparent in conventional seismic data. Petrophysical evaluation from well logs quantified key reservoir properties such as porosity, permeability, and hydrocarbon saturation, forming the foundation for static modeling. Structural and property modeling were integrated to construct a realistic three-dimensional reservoir framework and to distribute petrophysical parameters across the grid, improving the understanding of lateral and vertical heterogeneity. Facies modeling further identified sweet facies and potential new volumetric targets, while fault seal analysis evaluated the sealing capacity of major fault systems and their role in hydrocarbon entrapment. Volumetric calculations provided reliable reserve estimates, and uncertainty analysis was applied throughout the workflow to assess data sensitivity and ensure dependable interpretations. This integrated approach enhances confidence in reservoir characterization and provides a Robust foundation for future exploration and development of the Bahariya reservoir in the Berenice Field.

## Introduction

Recent advances in reservoir characterization have increasingly relied on the integration of seismic interpretation, petrophysical evaluation, and structural analysis to reduce subsurface uncertainty and improve hydrocarbon prediction. In complex clastic systems, particularly in structurally controlled basins, accurate delineation of reservoir architecture and fluid distribution requires combining multiple datasets, including well logs, seismic attributes, and geological models.

Seismic interpretation techniques have evolved significantly, enabling improved fault detection, stratigraphic delineation, and reservoir geometry reconstruction. Additionally, petrophysical modeling plays a fundamental role in quantifying reservoir properties such as porosity, permeability, water saturation, and lithology, which are essential for identifying hydrocarbon-bearing zones and estimating reserves. Recent studies have also emphasized the importance of fault seal analysis and structural validation in assessing reservoir compartmentalization and fluid migration pathways.

Despite these advancements, challenges remain in integrating these methodologies effectively, particularly in heterogeneous reservoirs where lateral facies variations and structural complexities significantly impact reservoir performance.

**Fig. 1:**
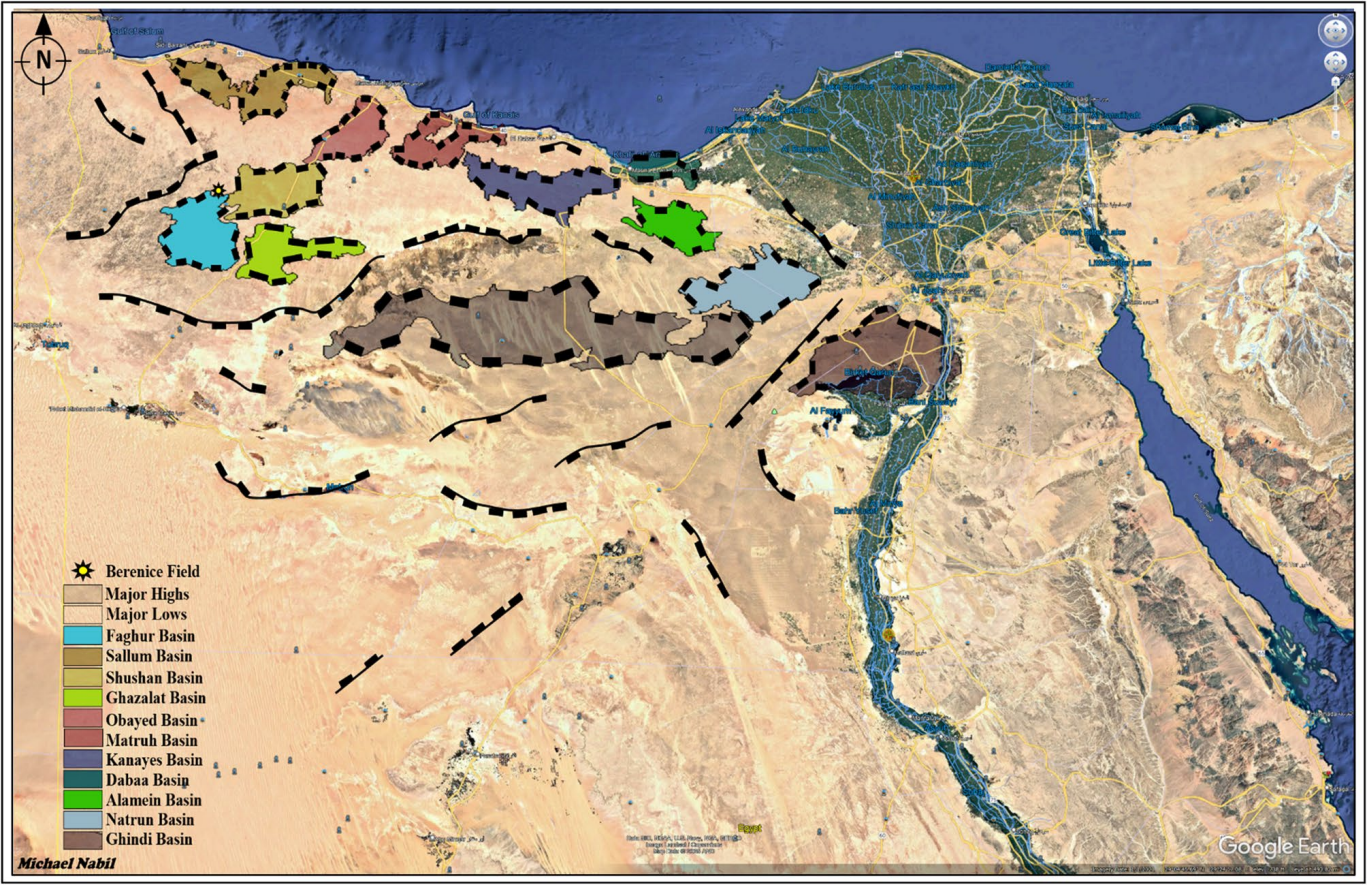
Simplified Geographic map of the North Western Desert of Egypt, highlighting major Stuctural Elements sedimentary basins, including the location of the Faghur Basin where the Berenice Field is situated. The Geographic Map was Generated using Google Earth Pro 7.3.6.10441. With Embedded Objects Using Petrel software, Version 2020.6 SLB. 15768.

The Bahariya Formation (BAH) in the North Western Desert of Egypt represents one of the most important Upper Cretaceous reservoirs, with proven hydrocarbon potential across several basins (Fig. 1)., including Shushan and Matruh. However, in areas such as the Berenice Field, detailed studies integrating seismic interpretation, petrophysical analysis, and structural evaluation remain limited.

Therefore, this study aims to provide an integrated reservoir characterization of the Bahariya Formation in the Berenice Field by combining seismic interpretation, petrophysical modeling, and structural analysis. The objective is to improve the understanding of reservoir heterogeneity, identify potential hydrocarbon zones, and evaluate the impact of structural features on reservoir performance.

**Fig. 2:**
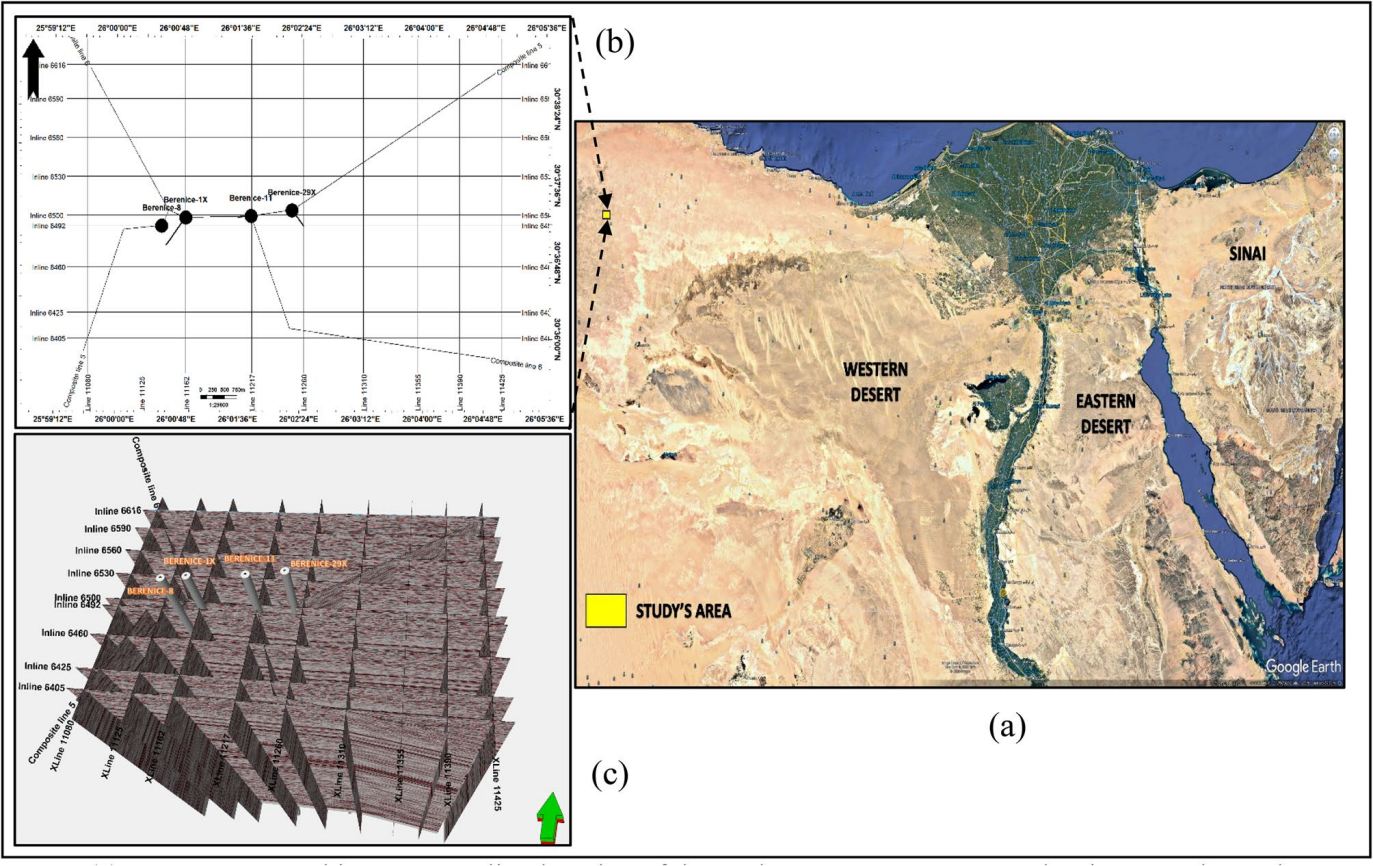
(**a**) Egypt’s geographic map revealing location of the study area. It was Generated Using Google Earth Pro 7.3.6.10441 (**b**) Base map showing the available seismic lines and wells location (**c**) The available Seismic lines in 3D domain, Base map and Seismic Lines Were Generated using Petrel software, Version 2020.6 SLB. 15768.

Among the productive regions within the Western Desert is the Berenice Field, located in the northeastern part of the Faghur Basin (Fig. 2). This field has gained attention due to its sustained hydrocarbon output, structural complexity, and well-established infrastructure. Its strategic location within a mature petroleum basin, coupled with high-quality reservoir rocks, positions it as a critical component of Egypt’s energy development plans. Continuous drilling and exploration efforts have revealed promising reservoir intervals, particularly within the Lower Cretaceous sequences, which have shown excellent reservoir potential and significant recoverable reserves.

## Geological settings

**Fig. 3:**
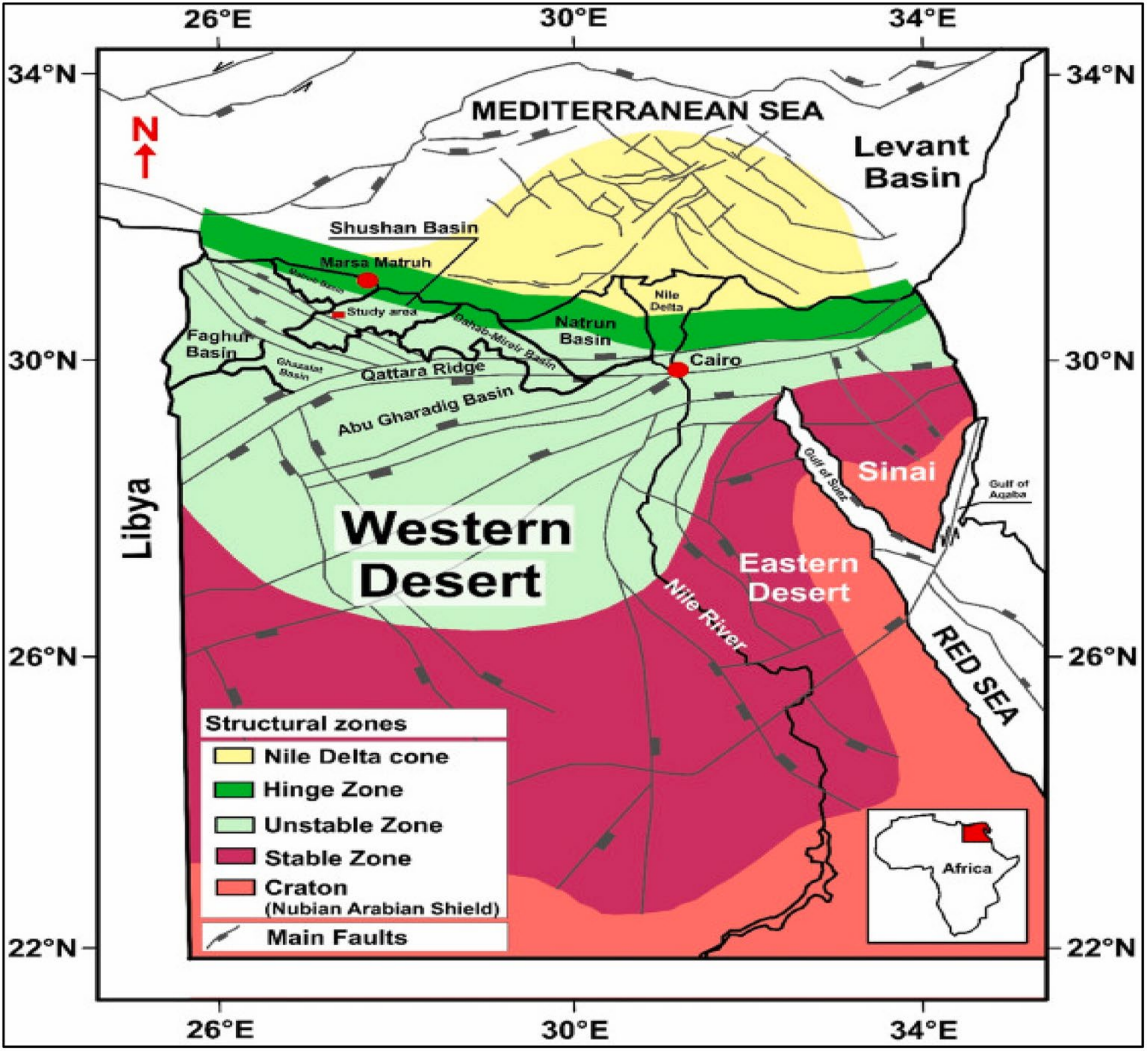
Sketch of main Tectonic Elements in Egypt’s Portion Shows The Following: Craton or Nubian Shield, Stable Zone, Unstable Zone, Hinge Zone and the Nile Delta Cone, This map is modified from WEC^13^, Meshref and El Sheikh^14^, Aal et al.^15^, Tari et al.^16^ and Ahmed I. Albrkawy et al. (2025). https://www.sciencedirect.com/science/article/pii/S0264817225001047#sec3.

Egypt is structurally subdivided from north to south into Four Segements (Fig. 3), the Miogeosyncline, Hinge Zone, Unstable Shelf, Stable Shelf, and Craton (Nubian–Arabian Shield)^1,2^ In the Western Desert, the sedimentary basins are divided into northern and southern provinces, separated by the E-W to ENE Ras Qattara–North Sinai uplift^3^.

Faghur Basin represents the western most mini-basin within the rift system of the Egyptian Western Desert. situated in the southern part of the Sallum Basin, Western Part of Shushan Basin and Northern Part of Ghazalat Basin in Egypt’s Western Desert (Fig. 2), has undergone a complex tectonic history influenced by multiple extensional and compressional phases^4^. This intra-cratonic basin developed primarily due to rifting events associated with the early Mesozoic break-up of Gondwana. The principal structural features consist of NE–SW and WNW–ESE oriented normal faults, which were subsequently superimposed or modified by transpressional deformation during the Late Cretaceous and Paleogene tectonic phases^5^. These tectonic regimes have influenced the basin’s segmentation, forming numerous fault-controlled traps that govern hydrocarbon distribution and migration routes^6^.

**Fig. 4:**
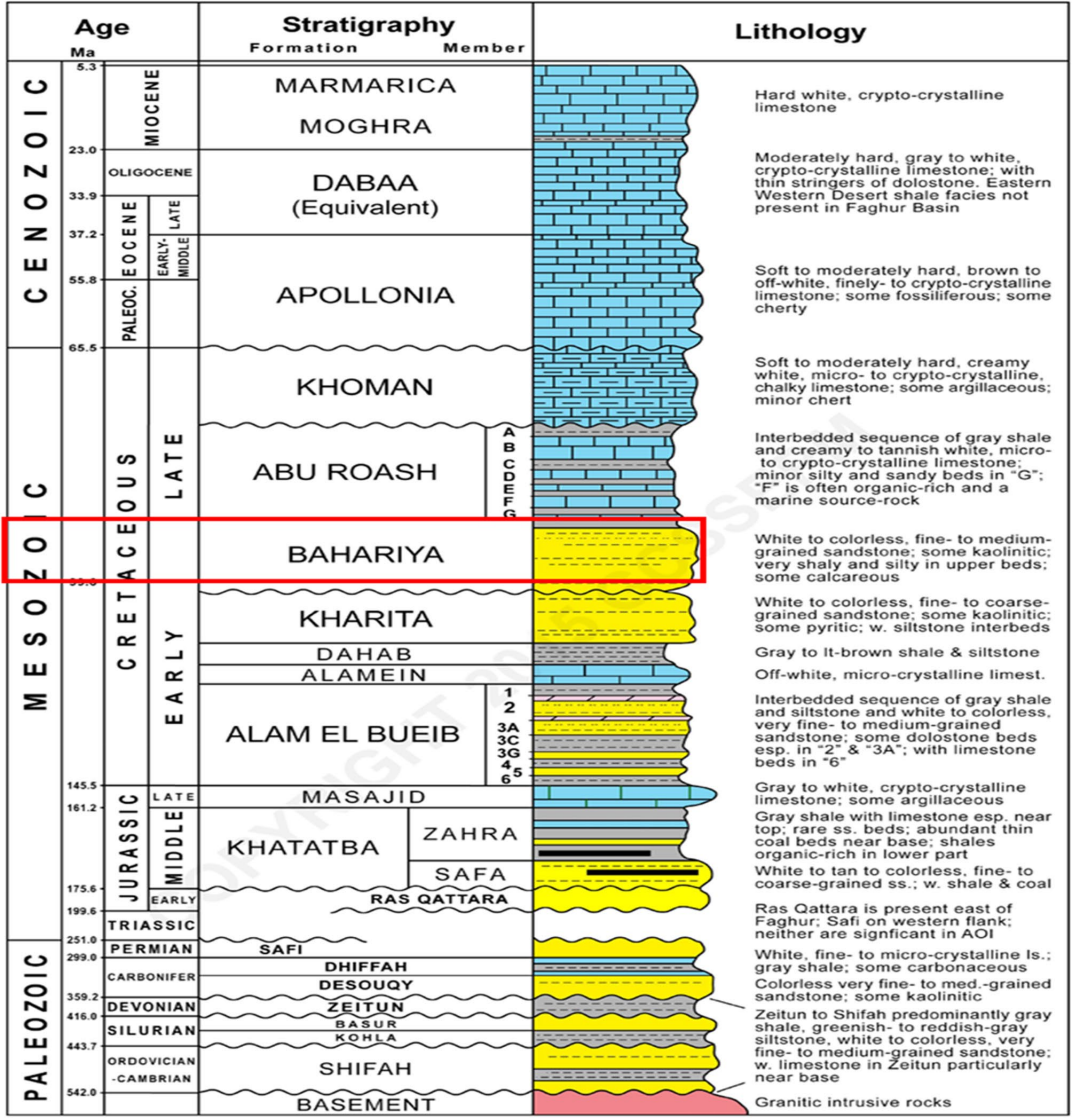
General stratigraphic column of Egypt’s north Western Desert^22^**.**

Stratigraphically, The Northern Western Desert hosts several Mesozoic basins containing thick successions and prolific hydrocarbon accumulations sourced mainly from Jurassic rocks^7^, notably in the Shoushan Basin^8,9^. The regional stratigraphy spans from Paleozoic to Cenozoic (Fig. 4), comprising four main depositional cycles: Lower–Upper Jurassic, Lower Cretaceous, Upper Cretaceous, and Eocene–Miocene^10–12^.

The Berenice Field, situated in the northeastern part of the Faghur Basin, occupies a region that has undergone notable tectonic uplift and subsidence events. The field exhibits a complex structural framework, characterized by tilted fault blocks that developed during Jurassic to Early Cretaceous rifting, and were later affected by inversion tectonics associated with the Syrian Arc.

compressional phase from the Late Cretaceous to Paleogene. The dominant fault orientations in the area trend NW–SE and NE–SW, mirroring the inherited rift geometry. These fault systems exert a significant influence on reservoir connectivity, fluid movement, and trap effectiveness, rendering structural interpretation essential for successful exploration and development operations within the field. Stratigraphically, The Northern Western Desert hosts several Mesozoic basins containing thick successions and prolific hydrocarbon accumulations sourced mainly from Jurassic rocks, notably in the Shoushan Basin^8^.

The regional stratigraphy spans from Paleozoic to Cenozoic (Fig. 4), comprising four main depositional cycles: Lower–Upper Jurassic, Lower Cretaceous, Upper Cretaceous, and Eocene–Miocene^10–12^.

The Precambrian basement of Faghur Basin, made up of igneous and metamorphic units, constitutes the structural core of the basin^17^. Overlying this foundation is a Paleozoic succession^18^, which is comparatively thin and weakly developed in the area. The Mesozoic sequence commences with the Jurassic Khatatba Formation, primarily consisting of alternating shale and sandstone layers, and is regarded as a key hydrocarbon source rock owing to its high organic richness^19^.

The Cretaceous succession is divided into a clastic Lower Cretaceous unit and a carbonate Upper Cretaceous unit^1^. Above it lies the Lower Cretaceous Alam El Bueib Formation, composed of sandstone, siltstone, and shale deposits, which serves as an important reservoir interval across numerous Western Desert fields, including the Berenice Field. The Kharita Formation (Albian) consists of quartzose sandstones with shale and siltstone interbeds, deposited in a shallow marine, high-energy environment and containing gas-prone carbonaceous shales^2^.

The overlying Bahariya Formation (Cenomanian) (zone of Study) comprises fine- to medium-grained quartzitic sandstones with minor shale and carbonate interbeds, deposited in a fluvial to shallow marine setting (Said, 1962;^20^). It serves as an important reservoir unit in the Western Desert.

Above it, the Abu Roash Formation represents a thick shallow-marine carbonate succession subdivided into seven members (A–G)^21^, containing type II kerogen with good source potential Definitely in Abu Roash F Member While the “G” Member often Provides an effective seal. The overlying Khoman Formation consists of massive chalky limestones deposited in an upper bathyal environment, acting primarily as a regional seal^2^ due to its low permeability.

The Paleocene to Eocene succession includes the Dabaa, Apollonia, and other formations that are mainly carbonates and marls, providing additional sealing capacity and overburden pressure for underlying reservoirs. The Paleocene to Eocene succession includes the Dabaa, Apollonia, and other formations that are mainly carbonates and marls, providing additional sealing capacity and overburden pressure for underlying reservoirs.

## Materials and methodology

**Fig. 5:**
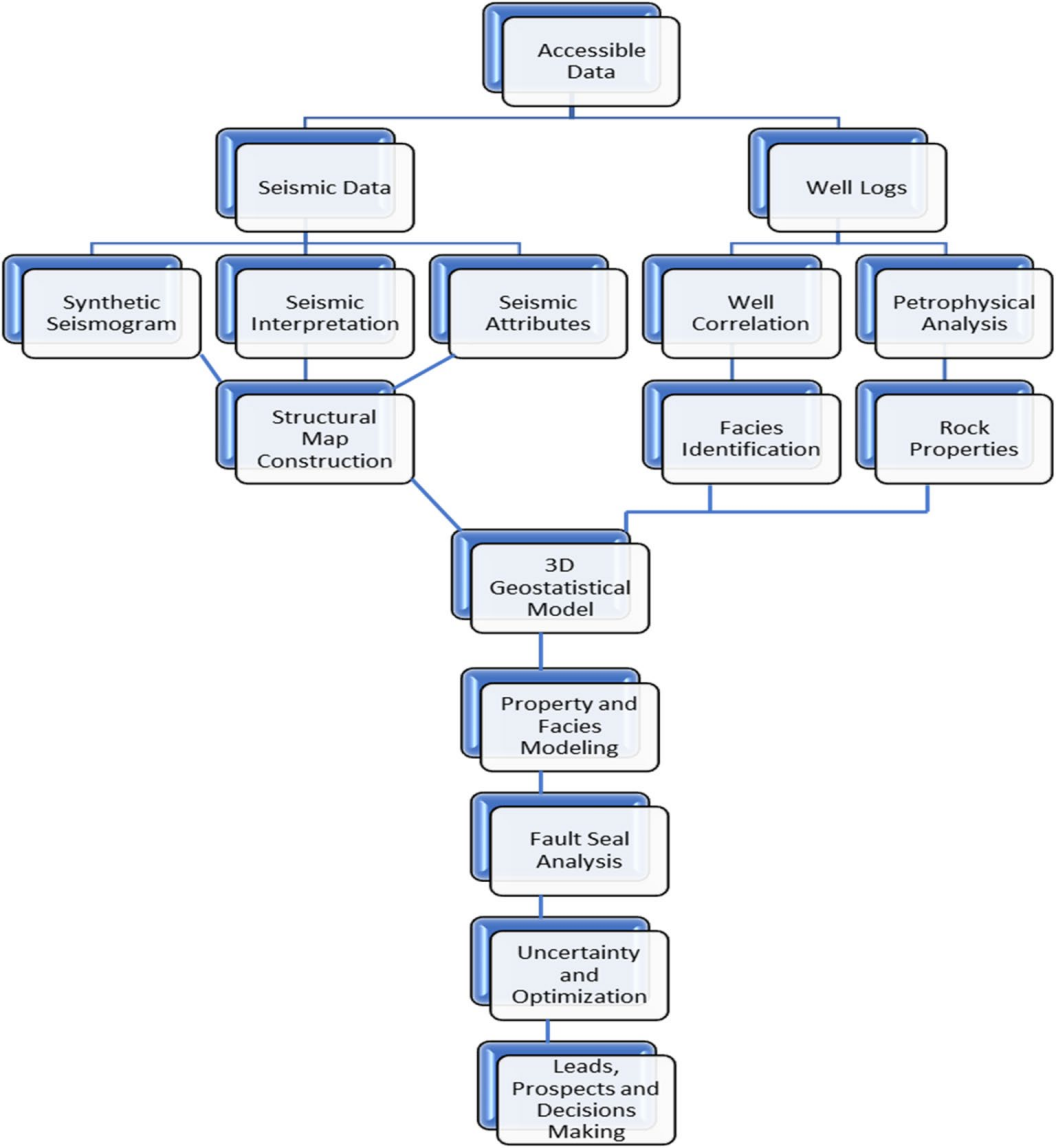
Shows the applied methodology for this study.

This study integrates geological, geophysical, and petrophysical datasets (Fig. 5) to construct a static reservoir model for the Upper Cretaceous Bahariya Formation in the Berenice Field, North Western Desert, Egypt. The available data include nine seismic inlines, nine crosslines, two arbitrary seismic profiles, and comprehensive Well Log Data.

Seismic interpretation was conducted to delineate major structural trends and map key horizons of the Bahariya Formation with Embedded Productive Units. Synthetic seismograms provided optimal well-to-seismic ties, ensuring accurate time–depth conversion. Seismic attribute analysis enhanced fault and fracture detection, defined stratigraphic orientations (dip and azimuth), and identified possible hydrocarbon indicators such as Bright spots.

**Fig. 6:**
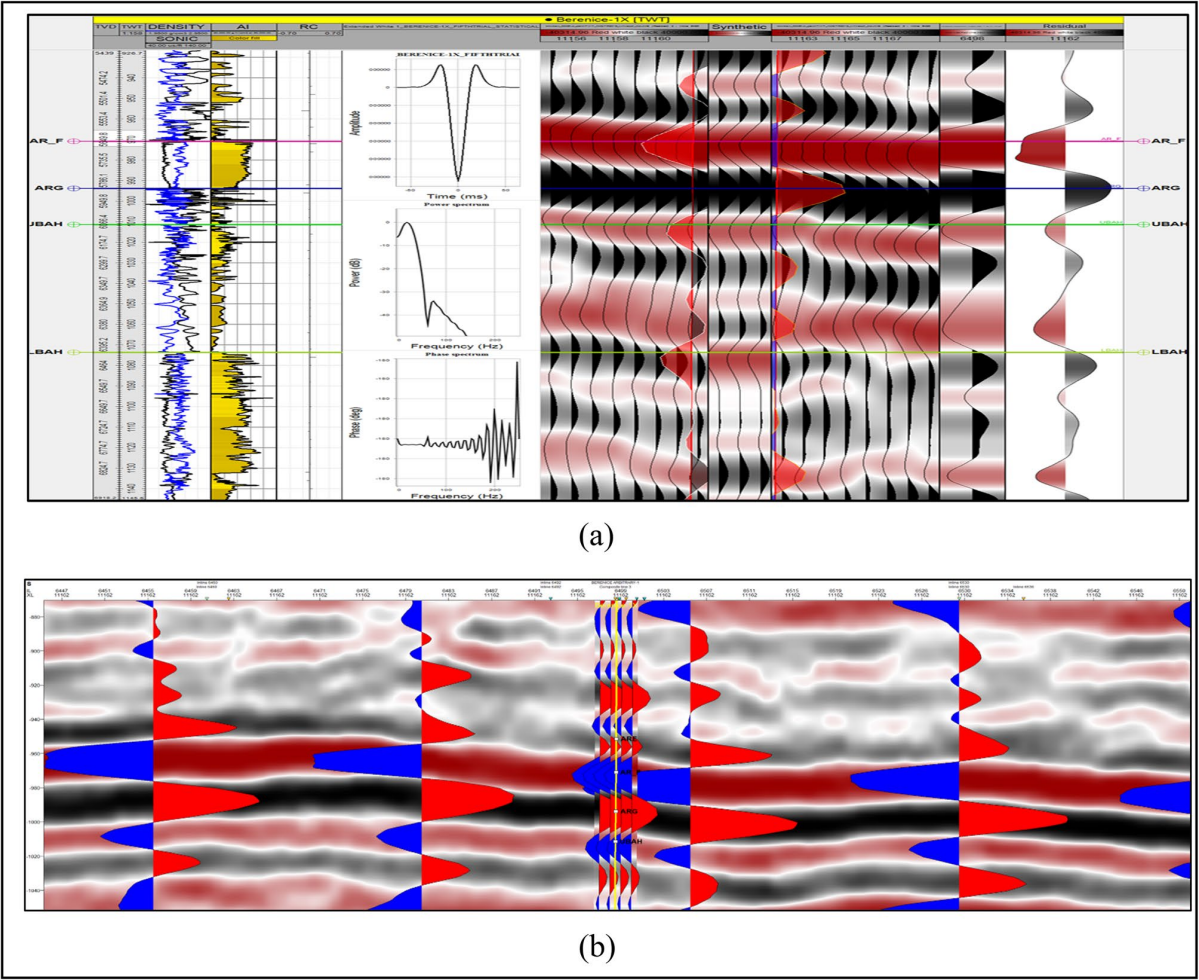
(**a**) A synthetic seismogram for Well BERENICE-1X, was created to correlate data of well log with the seismic section (**b**) Overlay for synthetic seismogram data on the seismic data.

Petrophysical evaluation was performed to determine porosity, permeability, fluid saturation, and facies distribution, supporting the identification of sweet spots and hydrocarbon-bearing zones^23^. A 3D static geological model integrated seismic and petrophysical results to visualize structural complexity and reservoir geometry^24^. Fault seal analysis assessed the sealing potential and compartmentalization of major faults. Reserves estimation applied volumetric methods to evaluate current and prospective reservoir zones. Finally, uncertainty analysis quantified parameter variability, improving confidence in hydrocarbon prediction and decision-making for field development (Fig. 6).

## Results and discussion

### Seismic data interpretation

**Fig. 7:**
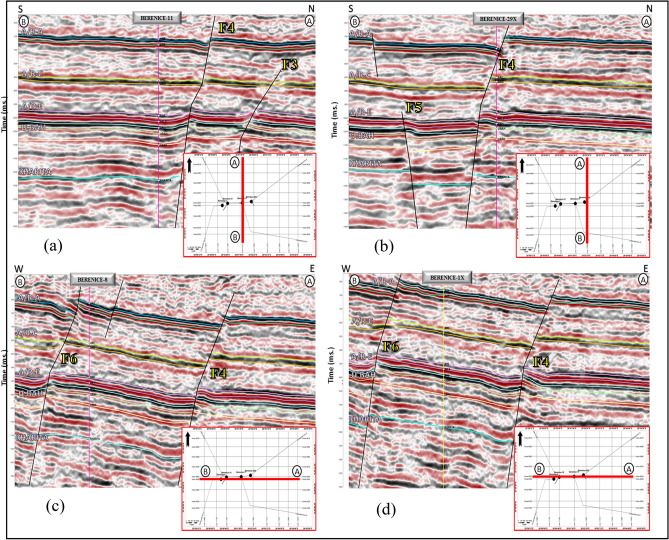
(**a**) Seismic section along line A-B passing through well Berenice-11, revealing the main interpreted stratigraphic horizons and normal faults F3, F4. (**b**) Seismic section along line A-B passing through well Berenice-29x, revealing the main interpreted stratigraphic horizons and normal faults F4, F5, where the well path intersects the major normal fault F4 with fault cut around 150 ft. (**c**) Seismic section along line A-B passing through well Berenice-8, revealing the main interpreted stratigraphic horizons and normal faults F4, F6. (**d**) Seismic section along line A-B passing through well BERENICE-1X, revealing the main interpreted stratigraphic horizons and normal faults F6, F4.

The interpretation of seismic data is influenced by both the characteristics of the dataset and the structural or stratigraphic methods employed^5^. A total of twenty seismic profiles (Fig. 7) were comprehensively analyzed to delineate the subsurface structural framework of the study area. The available well data were correlated with the 2D seismic sections to establish the reflector horizons corresponding to different stratigraphic intervals.

The dominance of NW–SE normal faulting suggests a structurally controlled reservoir system influenced by regional tectonic trends. These faults are likely to play a dual role, acting as both migration pathways and potential sealing boundaries depending on their throw and shale content. This structural framework directly impacts reservoir compartmentalization and hydrocarbon trapping efficiency.

### Synthetic seismogram generation

The importance of the synthetic seismogram lies in tying well data (in depth) to seismic data (in time), which enables recognition of seismic reflections that match geological formations. The sonic curve logged in the BERENICE-1X well was calibrated using a check-shot survey. The calibrated sonic log, together with the log of density and a statistical seismic wavelet, was then used to generate the synthetic seismogram by convolving the reflection coefficient log with the specified wavelet using Petrel™ (Version 2020.6). (Fig. 6) illustrates the correlation of the seismic cross sections with synthetic seismogram through well BERENICE-1X, marking the BAHARIYA members’ tops using the check-shot data^25,26^

The strong correlation between synthetic and seismic data significantly reduces time–depth uncertainty^27^, thereby improving the reliability of horizon picking and structural mapping. This enhanced calibration is critical for minimizing depth conversion errors, which directly influence volumetric estimations and reserve calculations.

### Seismic attributes

**Fig. 8:**
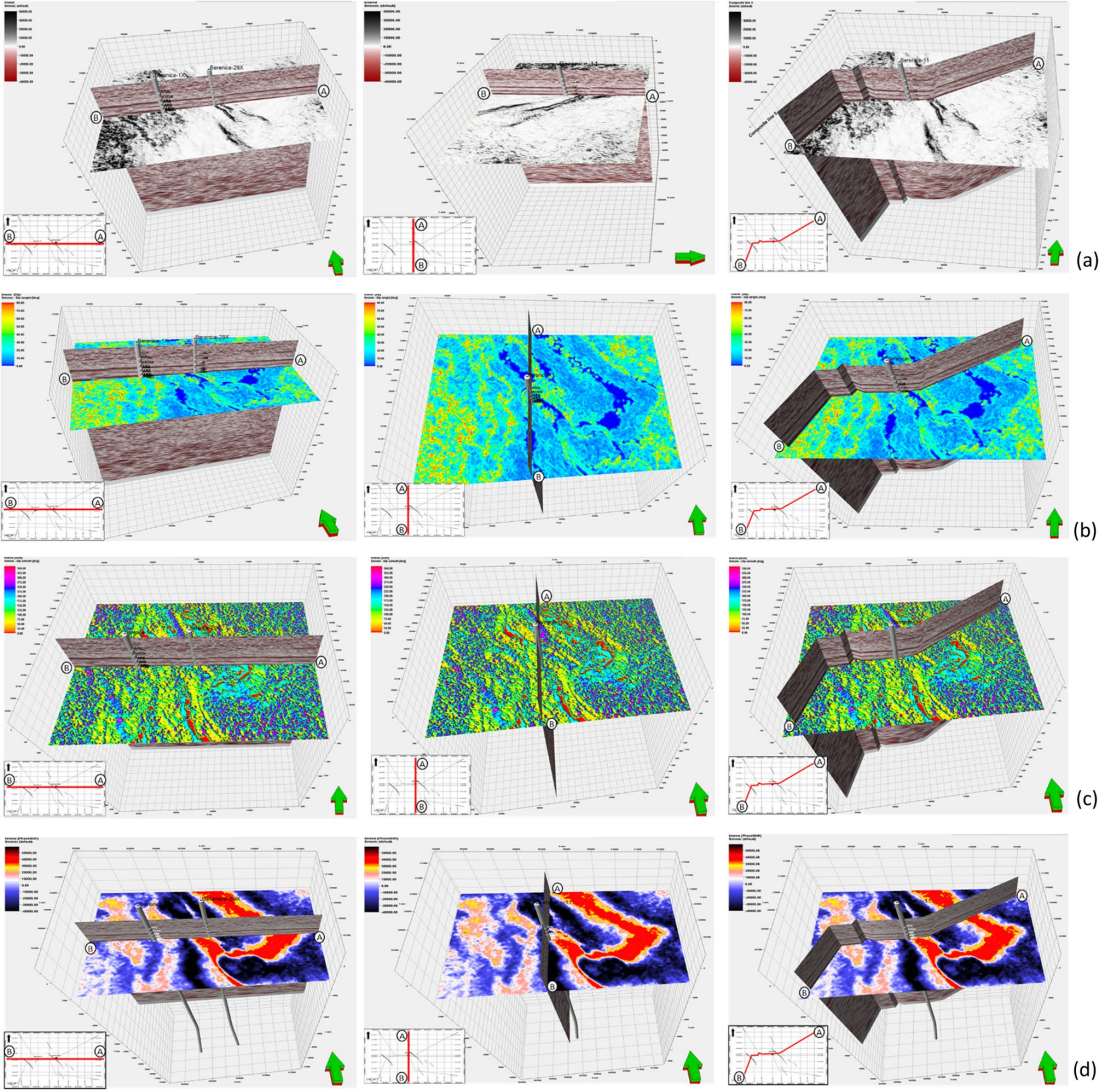
Seismic attribute panels showing: (**a**) variance for structural framework delineation, (**b**) local structural dip, (**c**) local structural azimuth, and (**d**) phase shift Used to Reveal phase anomalies and highlighting potential hydrocarbon-related phase changes.

Seismic attribute analysis was employed to enhance and Ensure the interpretation of the Bahariya Reservoir (Fig. 8), focusing on identifying structural configurations Throughout Bahariya Level and Also unveiling fault orientations, Attributes such as dip, azimuth, and amplitude Which were analyzed to delineate structural trends and dipping patterns^28,29^. These parameters also assisted in detecting hydrocarbon-bearing zones and Content through amplitude anomalies and reflection continuity variations^30^.

The detected amplitude anomalies, particularly within structurally elevated zones, are interpreted as potential direct hydrocarbon indicators (DHIs)^31^. When integrated with structural interpretation, these anomalies increase confidence in identifying prospective zones and reduce exploration risk^32,33^.

### Horizons and faults picking

Seismic lines crossing wells are commonly used as the first step in horizon identification as they allow for the correlation between the stratigraphy and seismic reflections^34^. Key horizons were picked to confirm local continuity and match properties. Horizons are usually interrupted by faults, making displacement^35^. The top of the Abu Roash F and Abu Roash G Member, a continuous high-frequency seismic reflector are identified across all seismic lines^36^. Additionally, key reservoirs in the Bahariya Formation Which are Represented in (Upper Bahariya Marker-1, Upper Bahariya Marker-2) were defined by picking the tops of units Upper Bahriya as shown in Fig. 7a–d.

In general, these N-S seismic sections display set of normal faults, affecting the Upper Cretaceous sequences with a downthrown side to the south West direction. In addition to that the Well (BERENICE-29X) was drilled to test the upthrown side of the North East normal fault Targeting the Bahariya reservoir.

### Structure maps construction

To explain the subsurface structure of the study area, the tops of three horizons (Upper Bahariya Top, Lower Bahariya Top and Kharita) were picked to create time structure contour maps then converted to depth domain using velocity maps^37^. The velocity model is applied to convert seismic data from time to depth domain. In this procedure, a seismic reference datum (SRD) is established at the beginning of the project. The applied method is the linear average velocity:

1 $${\mathrm{V}} = {\mathrm{V}}0 + {\mathrm{KZ}}$$

where V refers to the velocity at the datum and Z represents the vertical distance from the datum. V0 is the velocity at Z = 0, and will, typically much lower than the velocities within the subsurface layers. K is the gradient of velocity, ranging from 0 to − 0.2.

**Fig. 9:**
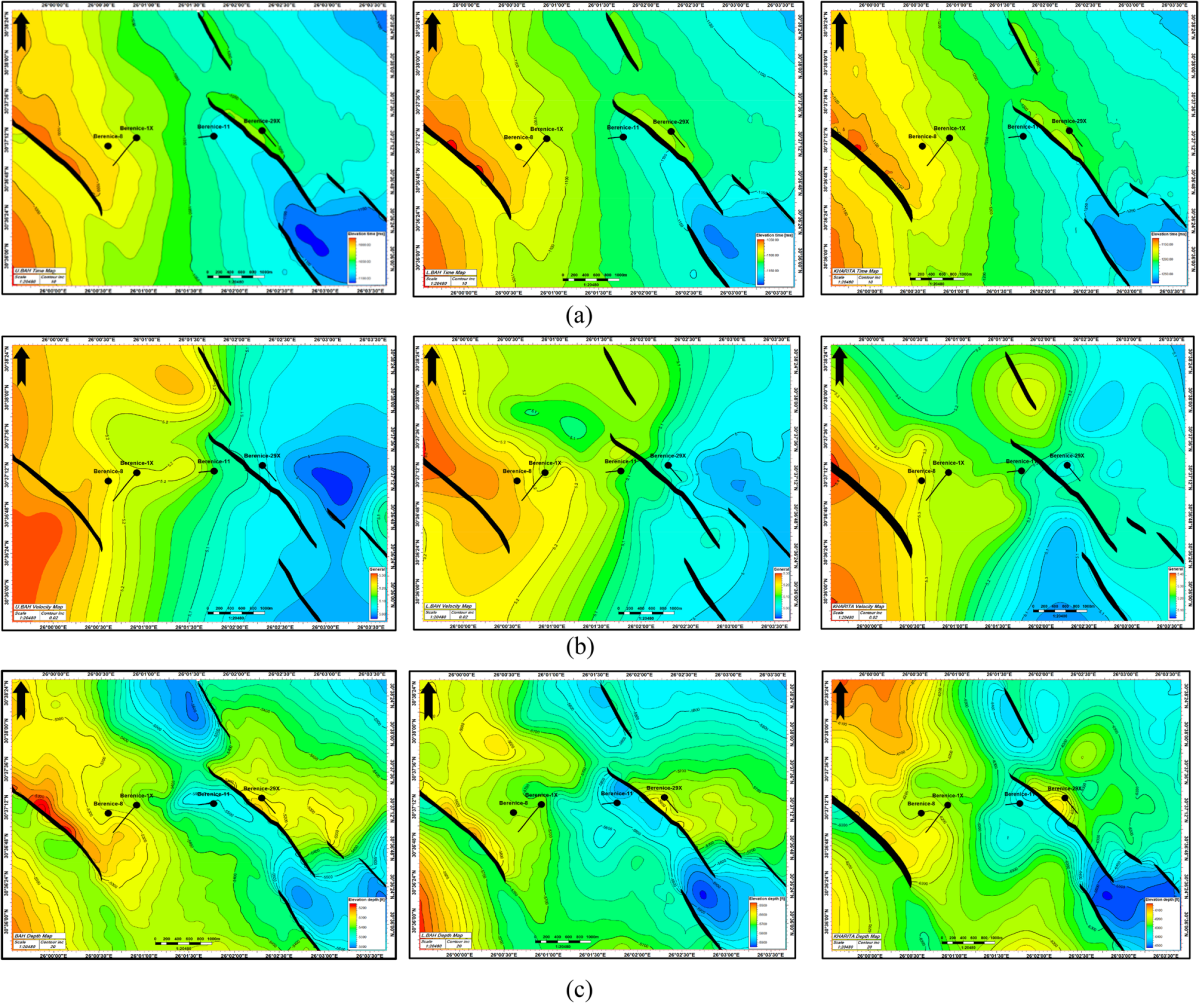
(**a**) Time, (**b**) interval velocity, and (**c**) depth-converted structural maps for the Upper Bahariya, Lower Bahariya, and Kharita horizons, illustrating the progression from seismic interpretation to depth-domain modeling.

The Depth Structure Contour Maps (Fig. 9) show patterns of normal faults with The Trend North West to South East (NW–SE) Direction. Whichs forms a three-way dip closure structure. This structural pattern offers the probability of hydrocarbon presence in high areas, particularly where faults are sealed. Table 1 highlights the summary of recognized probable prospects in the Berenice field, sorted by structural type and target reservoir.

**Table 1:** Volumetric reserve estimation summary for the eastern and western blocks.

Case	Area (acre ft)	Bulk Volume (10^3^) acre ft	Net Volume (10^3^) acre ft	Pore Volume (10^3^) acre ft	HCPV Volume (10^3^) acre ft	STOIIP (oil) (10^3^ STP)	Recovery factor %	Recoverable oil (10^3^ STP)
Eastern Blk	685	203	5	1	7787	7079	25	1770
Western Blk	262	78	12	3	17,945	16,313	25	4078

The identified three-way dip closures, combined with fault-assisted trapping mechanisms, indicate a structurally favorable environment for hydrocarbon accumulation. The effectiveness of these traps is strongly dependent on fault sealing capacity, emphasizing the importance of integrating fault seal analysis in reservoir evaluation.

### Petrophysical evaluation

The main purpose of petrophysical analysis is to retrieve useful data and information from the well logs^38–40^. The logs of gamma ray, resistivity, neutron and density were utilized to characterize the Bahariya Formation and its fluid saturation. Well production in hydrocarbon-bearing reservoirs is greatly influenced by key petrophysical properties like lithology, porosity, and water saturation. One wells’ electrical log in BERENICE field was analyzed for the Bahariya Formation. The volume of shale was determined by analyzing the data of Gamma ray log, which helps to differentiate between shaly and clean formations and quantify the content of shale in the reservoir. The shale volume (Vsh) can be calculated using the following equation:

2 $$V_{sh} = \left( {GR_{log} - GR_{min} } \right)/\left( {GR_{max} - GR_{min} } \right)$$

where V_sh_ and GR_log_ refer to the shale volume and the gamma ray reading from logs, respectively. GR_max_ and GR_min_ represent the maximum and minimum gamma ray readings, respectively.

The total porosity was estimated using the density and neutron logs, according to the following formula:

3 $$\emptyset_{T} = \, (\emptyset_{N} + \emptyset_{D} )/2$$

where ∅_T_ refers to the total porosity, while ∅_N_ and ∅_D_ represent the porosity derived from the neutron log, and the density log, respectively^41^.

The effective porosity was estimated using the following formula

4 $$\emptyset_{eff} = \emptyset_{T} - (V_{sh} * \emptyset_{sh} )$$

where ∅_eff_ denotes the effective porosity and ∅_T_ is the total porosity. Moreover, V_sh_, ∅_sh_ refer the shale volume, and shale porosity, respectively.

Water saturation was estimated by the following Indonesian equation, which assesses the percentage of the pore volume occupied by water.

5 $$\frac{1}{{\sqrt {R_{T} } }} = \left[ {\sqrt {\frac{{\phi^{m} }}{{aR_{{\mathrm{w}}} }}} + \frac{{v_{cl} \left( {\frac{{1 - v_{cl} }}{2}} \right)}}{{\sqrt {R_{cl} } }} } \right]s_{w}^{n}$$

where S_w_, n denotes water saturation and exponent of saturation, respectively. While R_t_ and V_cl_ refer to true resistivity and the volume of clay or shale, respectively. Moreover, ∅_T_ and ∅_sh_ represent the total porosity and shale porosity, respectively. Also, a, *R*_w_ and R_cl_ represent the tortuosity factor, resistivity of water formation, and the resistivity of clay or shale, respectively.

Beyond Saturation, Bulk Volume of Water (BVW) and Bulk Volume of Hydrocarbon (BVHC) were calculated to quantify the actual volume of fluids present within the reservoir rock. BVW is the product of effective porosity and water saturation, representing the volume of pore space filled with water. Conversely, BVHC represents the pore volume occupied by hydrocarbons and is derived by multiplying effective porosity by hydrocarbon saturation (S_h_). These volumes provide a direct measure of the fluid distribution within the reservoir, essential for estimating reserves and planning production. This can be calculated using the equation provided below

6 $$BVW = S_{W} *\emptyset_{eff}$$

7 $$BVHC = S_{HC} *\emptyset_{eff}$$

Permeability is a fundamental reservoir property that expresses the ability of a rock to transmit fluids through its interconnected pore system. In this study, permeability was estimated using empirical correlations derived from porosity and irreducible water saturation, which are routinely applied in clastic reservoir characterization. A widely used relationship is the Timur Equation, which relates permeability to porosity (ϕ) and irreducible water saturation (Sw_irr_) as:

8 $$k = 0.136\frac{{\phi^{4.4} }}{{S_{{w_{irr} }}^{2} }}$$

where $k$ = permeability (millidarcies, mD), ∅ = porosity (fraction or decimal), ${S}_{{w}_{irr}}^{2}$= irreducible water saturation (fraction).

Another commonly applied model is the Wyllie–Rose Equation, which expresses permeability as:

9 $$k = 7 \times 10^{3} \frac{{\phi^{6} }}{{S_{{w_{irr} }}^{2} }}$$

where $k$ = permeability (millidarcies, mD), ∅ = porosity (fraction or decimal), ${S}_{{w}_{irr}}^{2}$= irreducible water saturation (fraction).

For comparison and validation, permeability can also be approximated using the Kozeny–Carman Equation, which is more theoretical and relates permeability to pore geometry:

10 $$k = \frac{{\phi^{3} }}{{s^{2} \left( {1 - \phi } \right)^{2} }} \times \frac{1}{c}$$

where $k$ = permeability (millidarcies, mD), ∅ = porosity (fraction or decimal), ${S}_{{w}_{irr}}^{2}$= irreducible water saturation (fraction), S = specific surface area per unit grain volume (cm^2^/cm^3^).

C = Kozeny constant (dimensionless), describing pore-structure geometry.

**Fig. 10:**
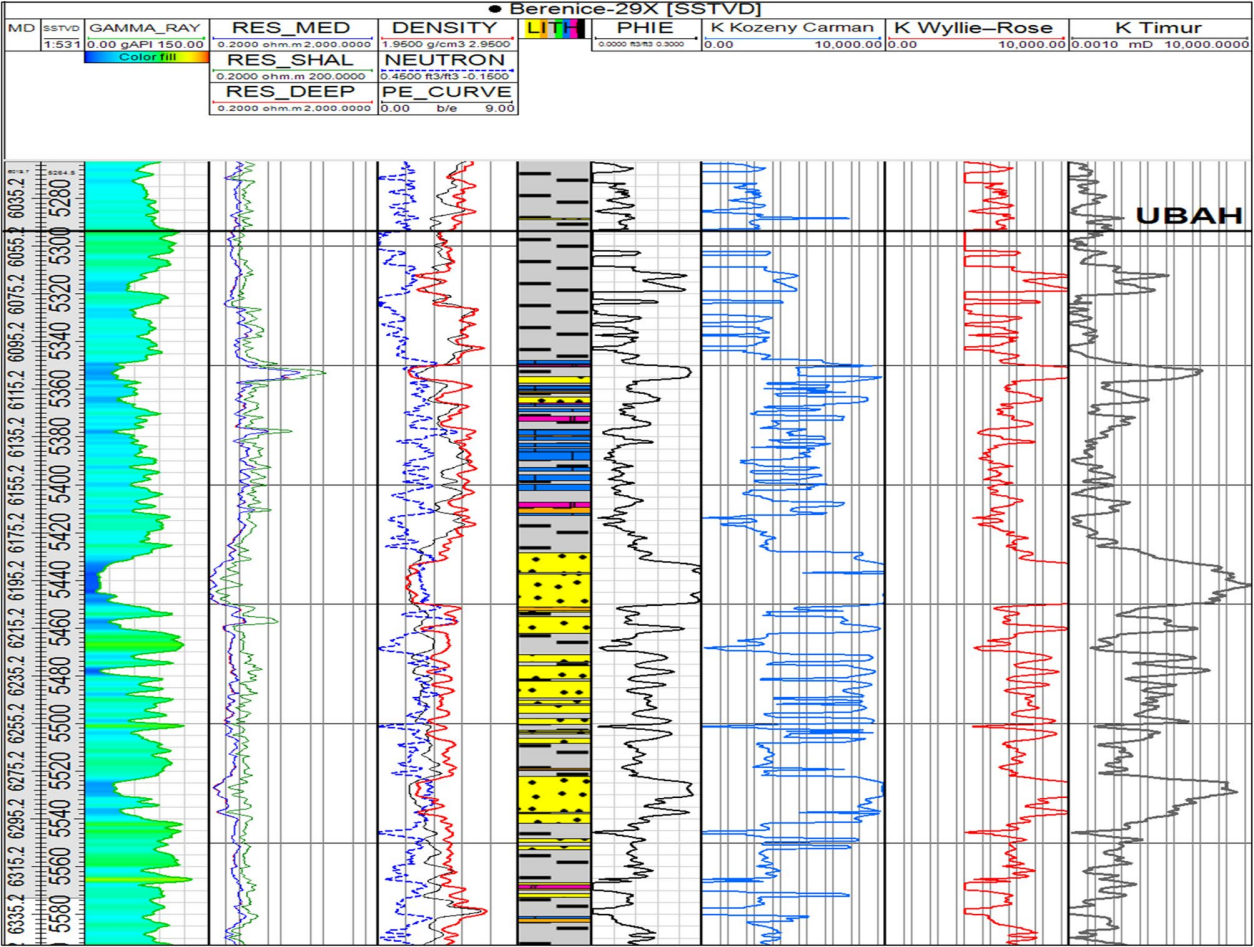
Wireline log Display Showing Calculated PHIE and permeability Estimates Derived From Kozeny–Carman, Wyllie–Rose and Timur Correlation For the Studied Well.

In this study, permeability was calculated using three commonly applied correlations—Timur, Wyllie–Rose, and Kozeny–Carman—to evaluate the full range of possible permeability behavior within the Bahariya reservoir^42^. While all methods were examined for comparison, the Kozeny–Carman equation was ultimately adopted as the primary permeability estimator. This selection was made because Kozeny–Carman is grounded in fundamental pore-scale flow physics rather than empirical fitting, provides more stable results in heterogeneous clastic systems, and better reflects the influence of pore geometry and specific surface area on fluid flow^43^. Accordingly, it was considered the most reliable approach for representing intrinsic reservoir permeability in the static model as Being Indicated in (Fig. 10).

The relatively high porosity values (26–27%) combined with low water saturation (24–25%) indicate favorable reservoir quality within the Upper Bahariya units^44^. However, the observed variability in shale volume highlights significant heterogeneity, which may adversely affect permeability distribution and fluid flow efficiency. This heterogeneity necessitates careful reservoir management and targeted development strategies^45^.

### Net-pay calculation

Net-pay estimation in the Berenice Oil Field was conducted by integrating petrophysical parameters with hydrocarbon indicators to define economically productive intervals. Cut-off values for porosity, water saturation, and shale volume were applied based on detailed evaluations. Techniques such as neutron-density crossover, resistivity separation, and movable hydrocarbon analysis ensured accurate identification of pay zones. The resulting net-pay interval (6067–6070ft), (6108–6115 ft) aligns closely with previously interpreted hydrocarbon-bearing zones Including Seismic Interpretation and Seismic Attributes. This consistency confirms the reliability of the applied methods and supports informed reservoir development planning.

**Fig. 11:**
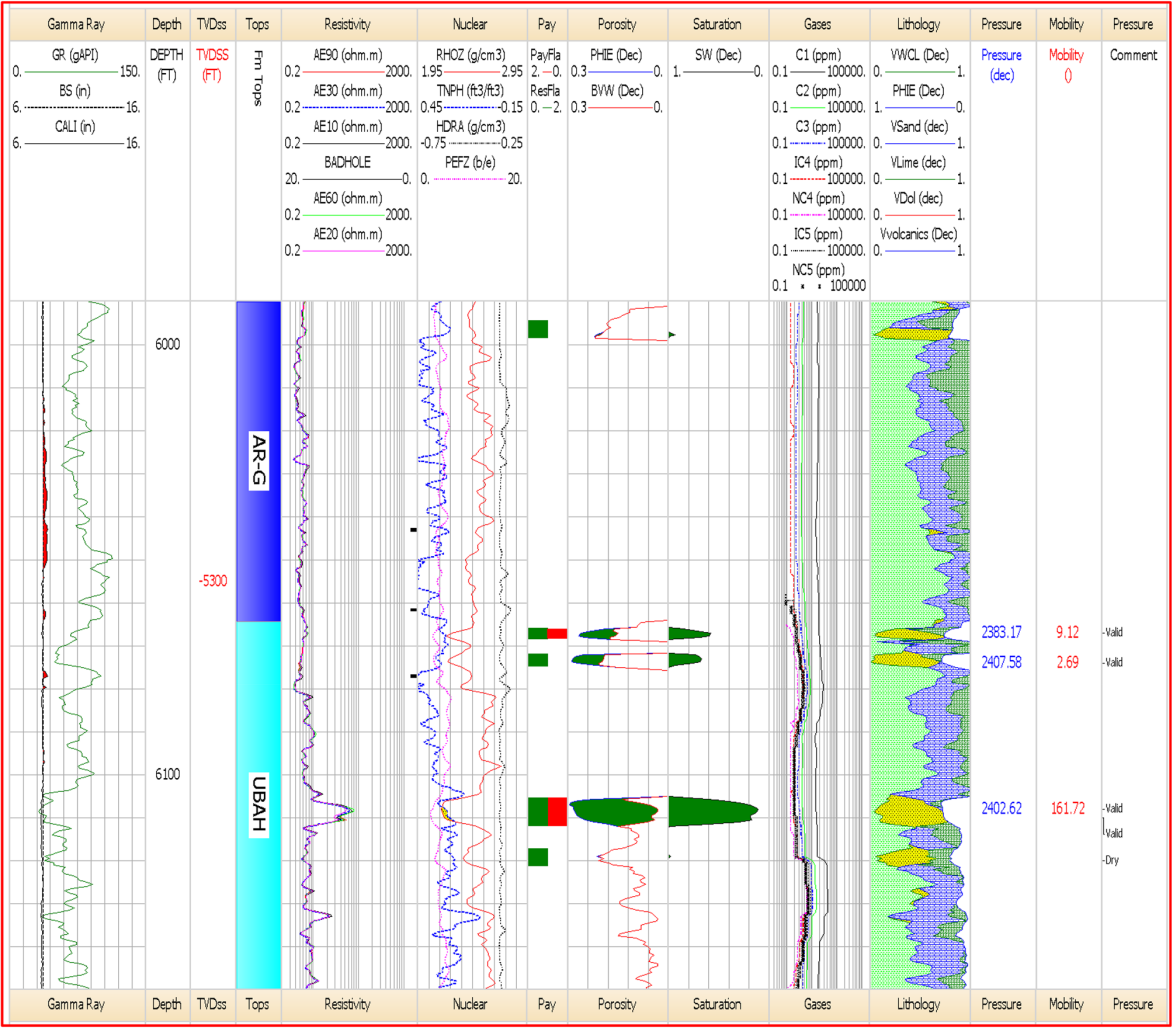
Litho-saturation plot for Well BERENICE-29X in the U.BAH Unit. The plot shows presence of 10 ft net pay, 26% average porosity and 25% average water saturation.

The petrophysical analysis results are summarized in Table 2. The positive results were obtained for units (Upper Bahariya Marker-1, Upper Bahariya Marker-2) in well Berenice-29X, as shown in (Fig. 11). For the Upper Bahariya Marker-1 unit, the net pay is 3 feet, with an average porosity of 27% and an average water saturation of 24% While Upper Bahariya Marker-2 Unit, The Net Pay is 7 feet, with an average porosity of 26% and an average water saturation of 25%.

**Table 2:** Shows loop and seed number with recoverable oil in each case.

Item	$Recoverable_ oil_10_3_STB_	$SEED	$LOOP
BERENICE_BAH_BLOCK29	3021.18397270233	20,015	37
BERENICE_BAH_BLOCK29	2865.51150705039	31,455	38
BERENICE_BAH_BLOCK29	1322.88823610221	11,034	39
BERENICE_BAH_BLOCK29	2530.13715805334	28,152	40
BERENICE_BAH_BLOCK29	1961.39105384589	1886	41
BERENICE_BAH_BLOCK29	1161.0762143958	16,152	42
BERENICE_BAH_BLOCK29	2694.73963867826	20,057	43
BERENICE_BAH_BLOCK29	3084.76642759286	30,392	44
BERENICE_BAH_BLOCK29	2900.3118045842	20,200	45
BERENICE_BAH_BLOCK29	2731.87882535752	5985	46
BERENICE_BAH_BLOCK29	1363.95593996907	15,368	47
BERENICE_BAH_BLOCK29	2731.59987331391	16,464	48
BERENICE_BAH_BLOCK29	3033.20351266019	22,381	49
BERENICE_BAH_BLOCK29	1616.67638919159	30,253	50
BERENICE_BAH_BLOCK29	2156.69528953982	15,833	51
BERENICE_BAH_BLOCK29	2110.05878551882	19,070	52
BERENICE_BAH_BLOCK29	2356.11360003941	19,267	53

The consistency between net-pay intervals and seismic-derived structural highs reinforces the reliability of the integrated workflow^46,47^. This agreement highlights the effectiveness of combining seismic interpretation with petrophysical analysis in reducing uncertainty in reservoir delineation^48,49^.

**Fig. 12:**
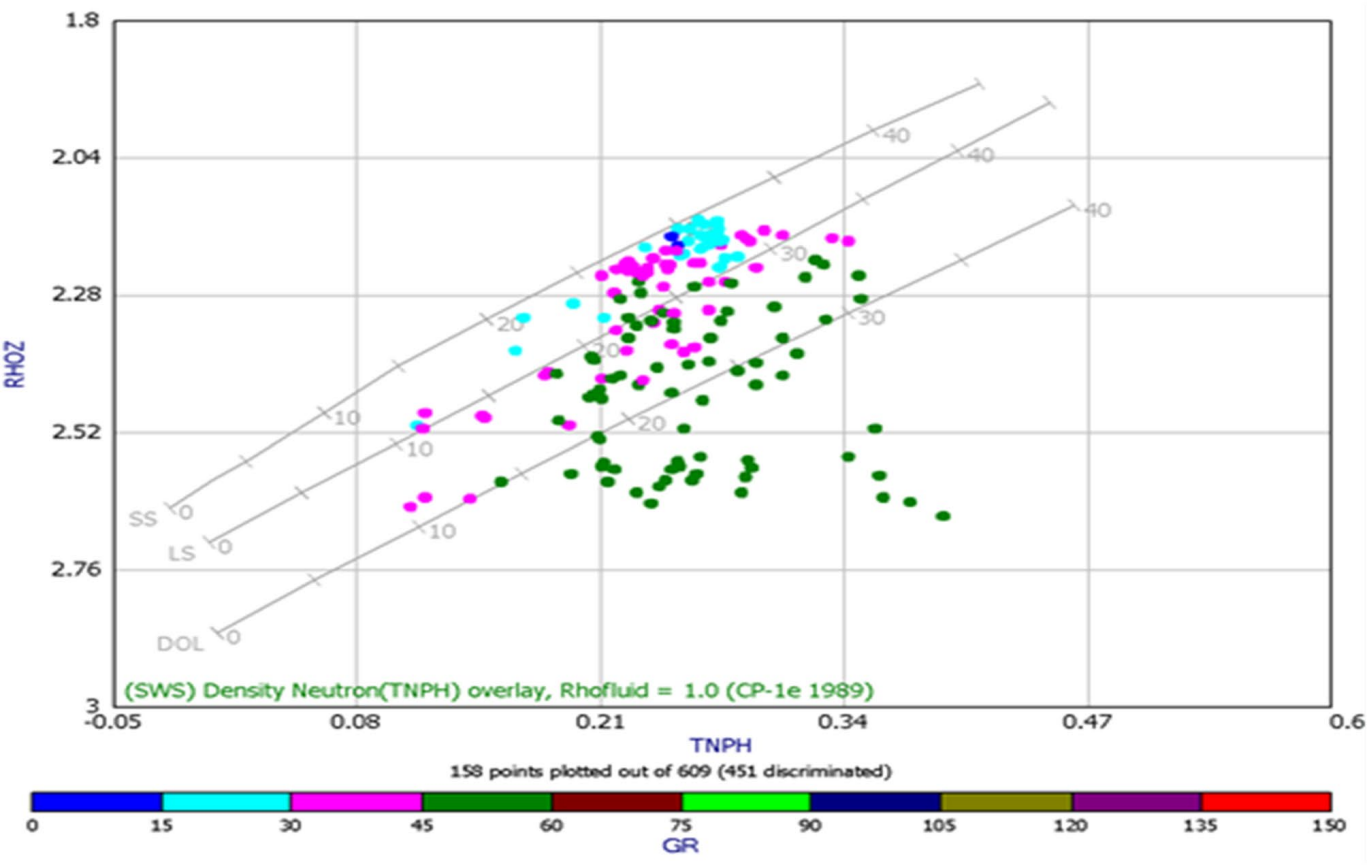
Density–neutron (RHOZ–NPHI) crossplot for the Bahariya Unit showing lithology trends, matrix composition, shale content and gamma-ray–based data distribution.

A density–neutron crossplot was generated for the Bahariya Unit as in (Fig. 12). to evaluate lithology and identify matrix trends within the studied interval. The plot integrates bulk density (RHOZ) and neutron porosity (NPHI) responses, allowing discrimination between sandstone, limestone, and dolomite fields using standard lithology trend lines^50^. Data points showed a dominant clustering toward the sandstone trend, indicating siliciclastic composition with limited carbonate influence. The color coding represents gamma-ray values, enabling quick differentiation between clean and shaly intervals. This crossplot provides a robust lithological framework that supports subsequent porosity and permeability evaluations for the Bahariya reservoir.

In General The petrophysical analysis indicates that the structural configuration, particularly the proximity to faults, likely plays a significant role in controlling fluid distribution and reservoir quality. The Bahariya Formation is characterized by sand lenses interbedded with shale and siltstone layers, exhibiting marked heterogeneity in reservoir properties across the field. Nevertheless, the seismic interpretation reveals promising indicators that may help unlock hidden potential and subtle reservoir features within the formation.

### 3D modeling

**Fig. 13:**
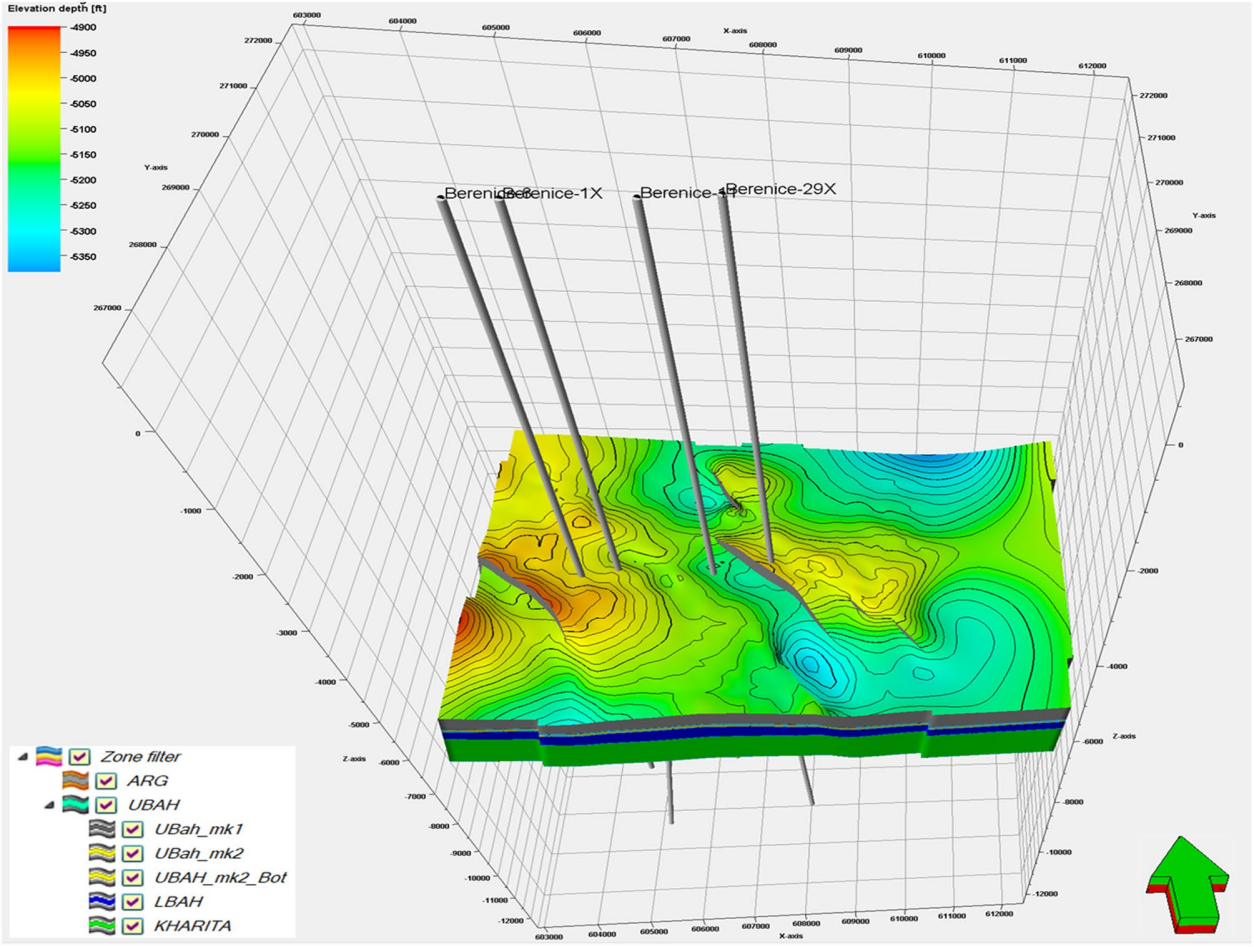
Shows 3D depth structural model of the Berenice field Revealing, faults geometry, surfaces, zones, and layers. The model combines interpretation of seismic and well log data to provide a picture for structural features in three dimensions.

Integrating multiscale datasets seismic data for regional structural context and well logs for high-resolution reservoir characterization enhances the accuracy of delineating the Upper Cretaceous reservoir architecture, identifying heterogeneities in porosity, lithology, and fluid distribution, and reducing uncertainty in the geological model^51–53^. The modeling workflow comprises structural, petrophysical, and facies modeling. Structural modeling establishes the 3D framework by defining the model geometry, faults, and horizons. The model area is first outlined using seismic survey coverage, followed by fault modeling based on depth-converted seismic interpretations. A 2D pillar grid is then constructed in Petrel™ (2020.6). Horizon modeling incorporates depth maps of the Abu Roash G Member and top Bahariya surface, with thickness maps and formation tops used to build conformable internal zones: Upper Bahariya Marker-1, Upper Bahariya Marker-2, Lower Bahariya, and the Kharita Formation. The resulting 3D structural model (Fig. 13) shows that Bahariya units generally dip northeastward, with low-relief areas in the southeast and north, and higher-relief zones toward the east, west, northwest, and southwest.

The 3D structural modeling delineates two prominent four-way dip closures within the study area, the first situated in the northwestern sector and the second in the southwestern sector. These closures represent structurally favorable traps that merit further evaluation, particularly in light of the seismic interpretation and attribute analyses, which revealed notable amplitude anomalies indicative of potential hydrocarbon accumulations. The observed amplitude variations across these closures suggest promising subsurface conditions, emphasizing their significance as priority targets for subsequent exploration and appraisal activities^54^.

The newly constructed 3D geological model^55^ provides a comprehensive visualization of the spatial distribution of key reservoir properties within the Bahariya Formation as in (Fig. 13). Effective porosity (PHIE) is clearly differentiated across the model, highlighting zones of enhanced reservoir quality where cleaner, better-connected pore systems dominate Especially Near Closure Where BERENICE-29X is Located and Recorded 26% and The Western Closure Where BERENICE-1X is Located Nearby. Shale volume (Vsh) mapping and Distribution delineates intervals of heterogeneous lithology, enabling recognition of laminated versus cleaner sand bodies that directly influence petrophysical behavior and Sort the Sand Quality Whereas BERENICE-29% Exhibited a Shale Volume Value of 15%, also it’s worth to be mentioned that A noticeable reduction in shale volume is observed toward the western structural closure, indicating the presence of cleaner reservoir intervals. These low-Vsh zones enhance reservoir quality and promote this area as a potential hydrocarbon trapping hotspot. Which is Compatible with Seismic Attribute’s Outcomes.

**Fig. 14:**
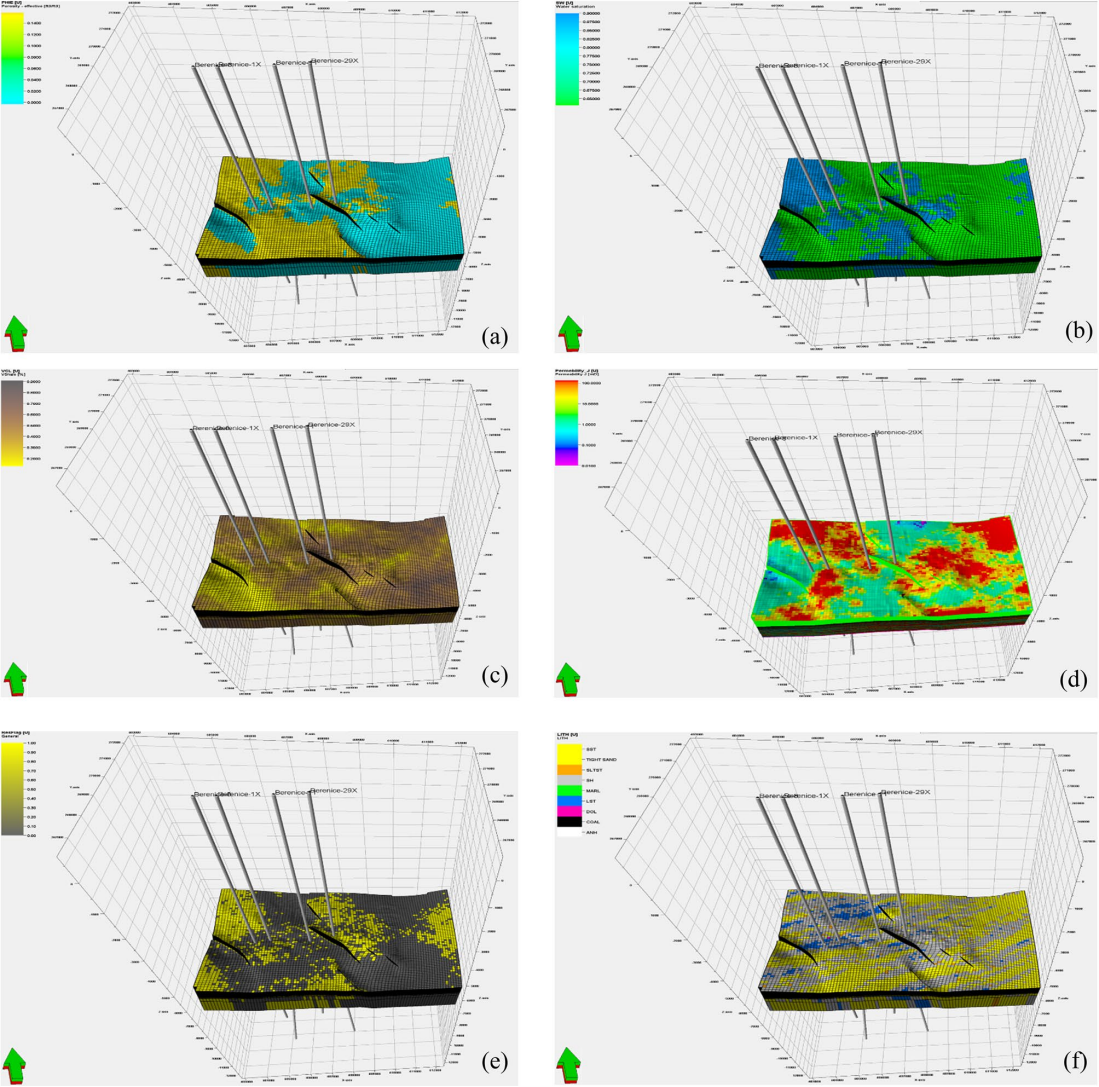
Integrated 3D geological model illustrating Spatial distribution of key reservoir properties: (**a**) Porosity: PHIE (**b**) Water Saturation : Sw, (**c**) Shale Volume :VCL, (**d**) Permeability : K (**e**) Net Gross : N/G phase (**f**) Facies and Lithology : LITH.

Water saturation (Sw) distribution reveals Distinct fluid contacts and saturation gradients, that reflect the heterogeneous nature of its sand facies and variable reservoir quality. These heterogeneities result in uneven fluid distribution throughout the field. On other side Water Saturation (Sw) in BERENICE-29 was Measured at 25%. and Notably, the model indicates consistently lower water saturation values toward the western structural closure, reinforcing the interpretation of enhanced hydrocarbon potential in this area and supporting its prioritization as a key exploration target. Permeability modeling populated using the Kozeny–Carman relationship captures lateral and vertical variations in flow capacity, emphasizing high-quality channels Represented in Upper Bahariya Marker-1 Whereas Permeability Average Values was Measured 240 Milli Darcy and also permeability shows Promising Values Near the Western Segment of BERENICE Field Which further substantiating the presence of favorable hydrocarbon conditions and justifying its elevation as a primary exploration focus. Net-to-gross (N/G) ratios further distinguish reservoir-prone intervals from non-reservoir layers, providing an integrated view of sand-body continuity and thickness. Facies distribution, derived from well data as Shown in (Fig. 14). and propagated through the 3D grid, illustrates the arrangement of depositional elements and highlights the lateral extent of sweet facies Especially Sand Facies with the greatest reservoir potential. Collectively, these properties allow for a more accurate characterization of reservoir heterogeneity and significantly enhance confidence in volumetric estimation and development Strategies also Serve to Mitigate the Drilling Hazards.

The integration of seismic, petrophysical, and structural data within a unified 3D framework significantly enhances the prediction of reservoir heterogeneity and fluid distribution^56–60^. This approach not only improves volumetric accuracy but also provides a robust platform for future dynamic simulation and field development planning.

**Fig. 15:**
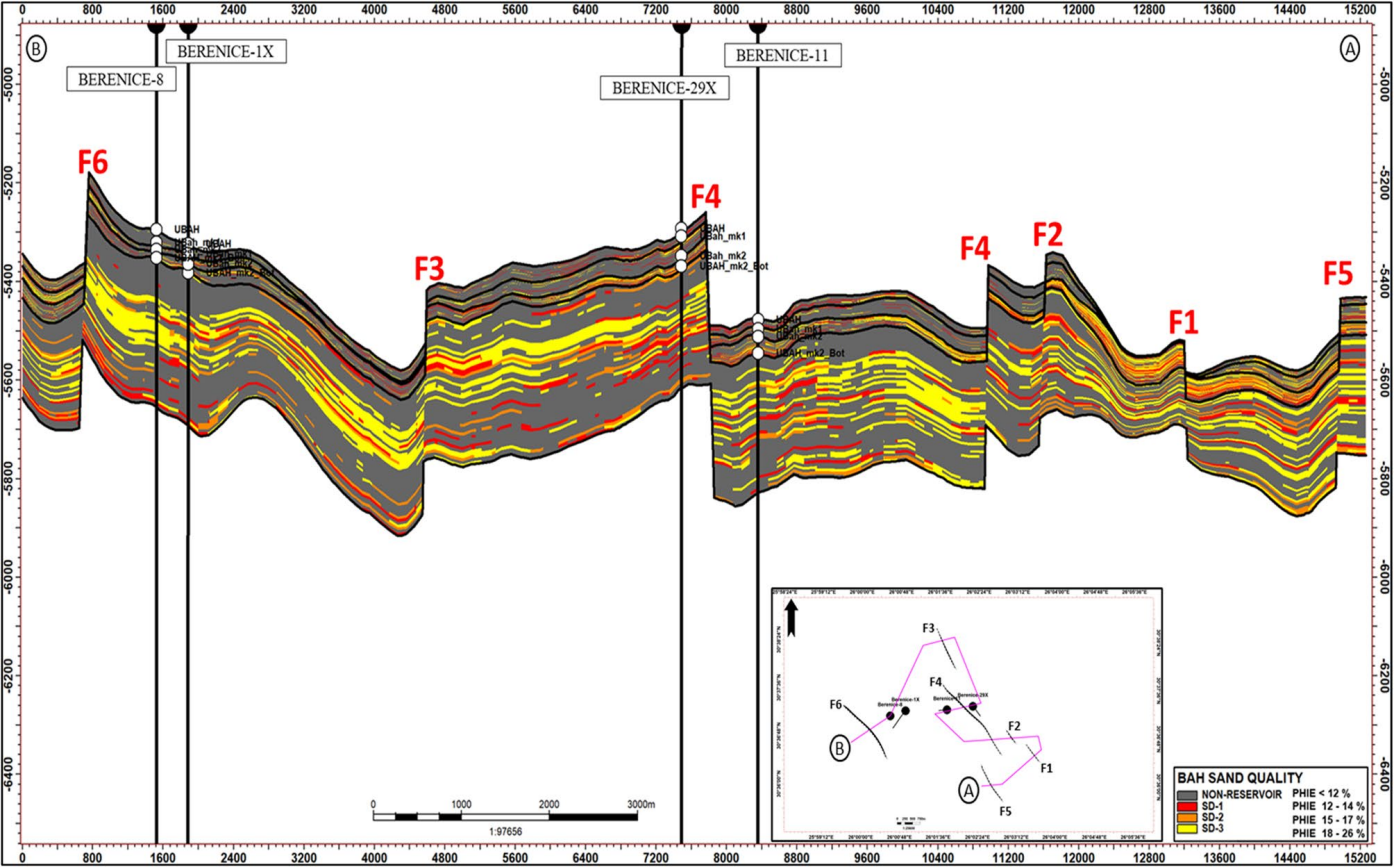
Integrated cross section (**A**–**B**) passing through wells BERENICE-11, BERENICE-29X, BERENICE-1X and BERENICE-8. The section displays lateral variations in reservoir thickness, Reservoir Sand Quality showing that the area is bound by a Three major normal faults F3, F4, F6 and Three Minor Ones F1, F2, F5 and BERENICE-29X was drilled on the up-thrown side of Major Fault F4.

The cross-section (A–B) passing through the BERENICE wells (Fig. 15) demonstrates that the three-dimensional structural model has been robustly validated by aligning modeled horizons with well tops from existing wells, ensuring that the model accurately reflects formation depths and key structural features at each well location. Noteably that The field is structurally bounded by three major normal faults and three minor ones of NW- SE Orientation, Importantly, this cross-section not only illustrates the structural framework and formation boundaries but also highlights variations in sand quality and reservoir grading throughout the Bahariya Formation, offering insights into lateral and vertical heterogeneity.

**Fig. 16:**
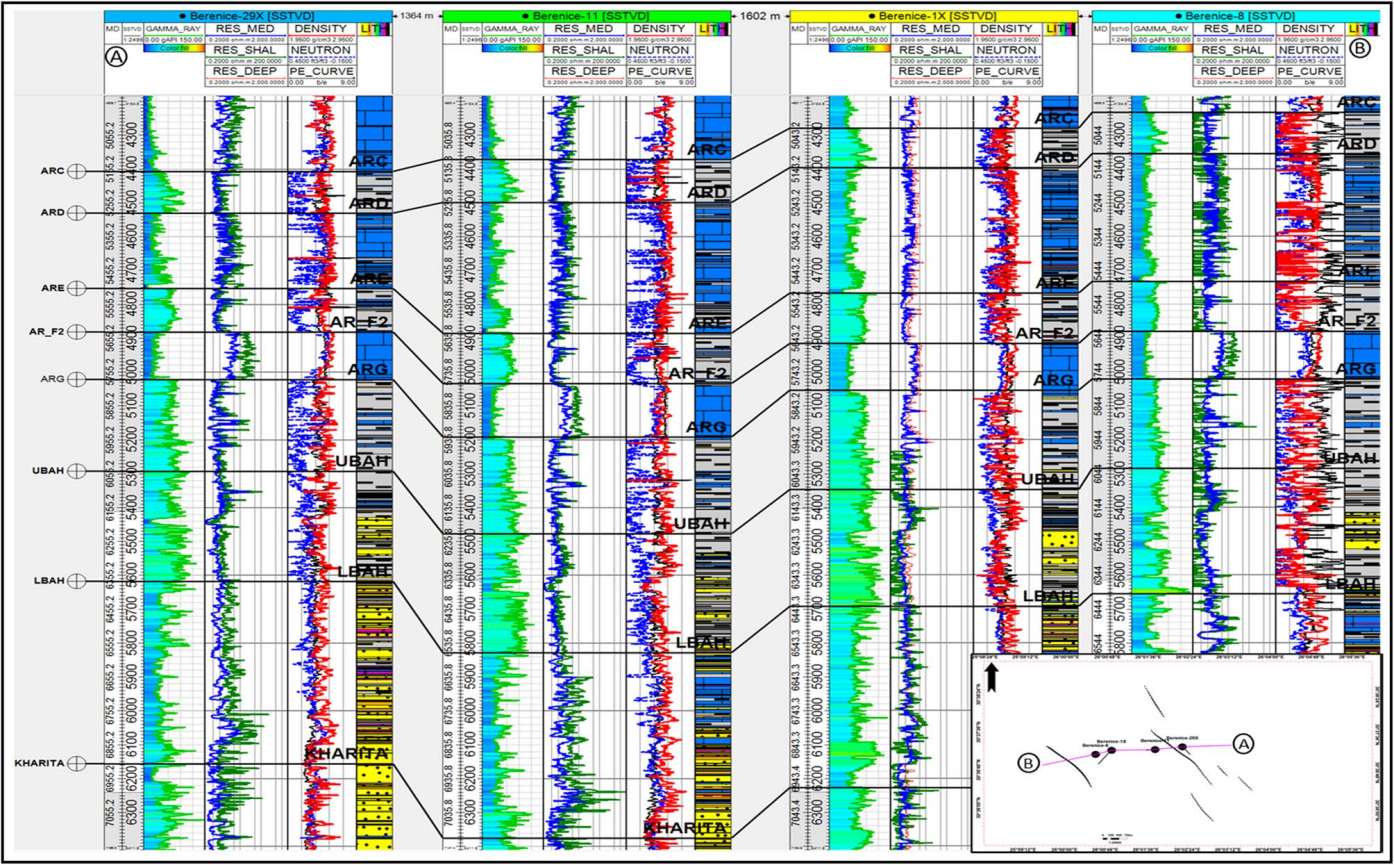
A structural well correlation along the line (**A**–**B**) in the study area shows changes in lithology and well log Responses, Also Correlation shows a slight thinning of the Abu Roash D Unit toward the east, attributed to the intersection of the BERENICE-29X Well trajectory with Major Fault F4, which results in an apparent missing section of approximately 150 ft.

Additionally, the well-to-well correlation as Shown in (Fig. 16) along this section enhances understanding of stratigraphic continuity, sand-body connectivity, and facies distribution across the field. Correlating these wells provides further evidence for the consistency of reservoir architecture and supports the reliability of the constructed geological model^61^.

### Modeling uncertainties and limitation

The building of the 3D geological model is influenced by the following main factors: The limited vertical and lateral resolution of seismic data increases uncertainty in identifying horizons and faults^62^. Seismic resolution below Abu Roash G may need to be improved through advanced reprocessing techniques or by acquiring new seismic data with updated acquisition parameters. The limited wells number in the study area decreases confidence in seismic-to-well ties.

To reduce this uncertainty, specifically in the southern, South West and Western parts of the study area, additional wells with Vertical Seismic Profiling (VSP) data may be required. Lateral velocity changes, resulting from structural and facies variations, lead to errors in depth estimation, which may affect the accuracy of identifying structural geometry and volumetric calculations. The structural model is static and lacks geo-mechanical data, such as in-situ stress^63^. Moreover, the absence of dynamic data, like production data and 4D seismic, decreases the ability to validate and predict reservoir performance.

### Fault seal analysis

Fault seal analysis in this study is essential for assessing the ability of faults to restrict or permit hydrocarbon flow^64–67^. The evaluation incorporates key parameters such as Transmissibility Multiplier (T), Shale Gouge Ratio (SGR), fault geometry, displacement, heave, thickness, and fault-zone permeability. Together, these factors define fault sealing efficiency and reservoir compartmentalization. In the Bahariya Formation, the integration of these properties indicates that most faults exhibit low transmissibility and high sealing capacity, particularly across hydrocarbon-bearing intervals.

**Fig. 17:**
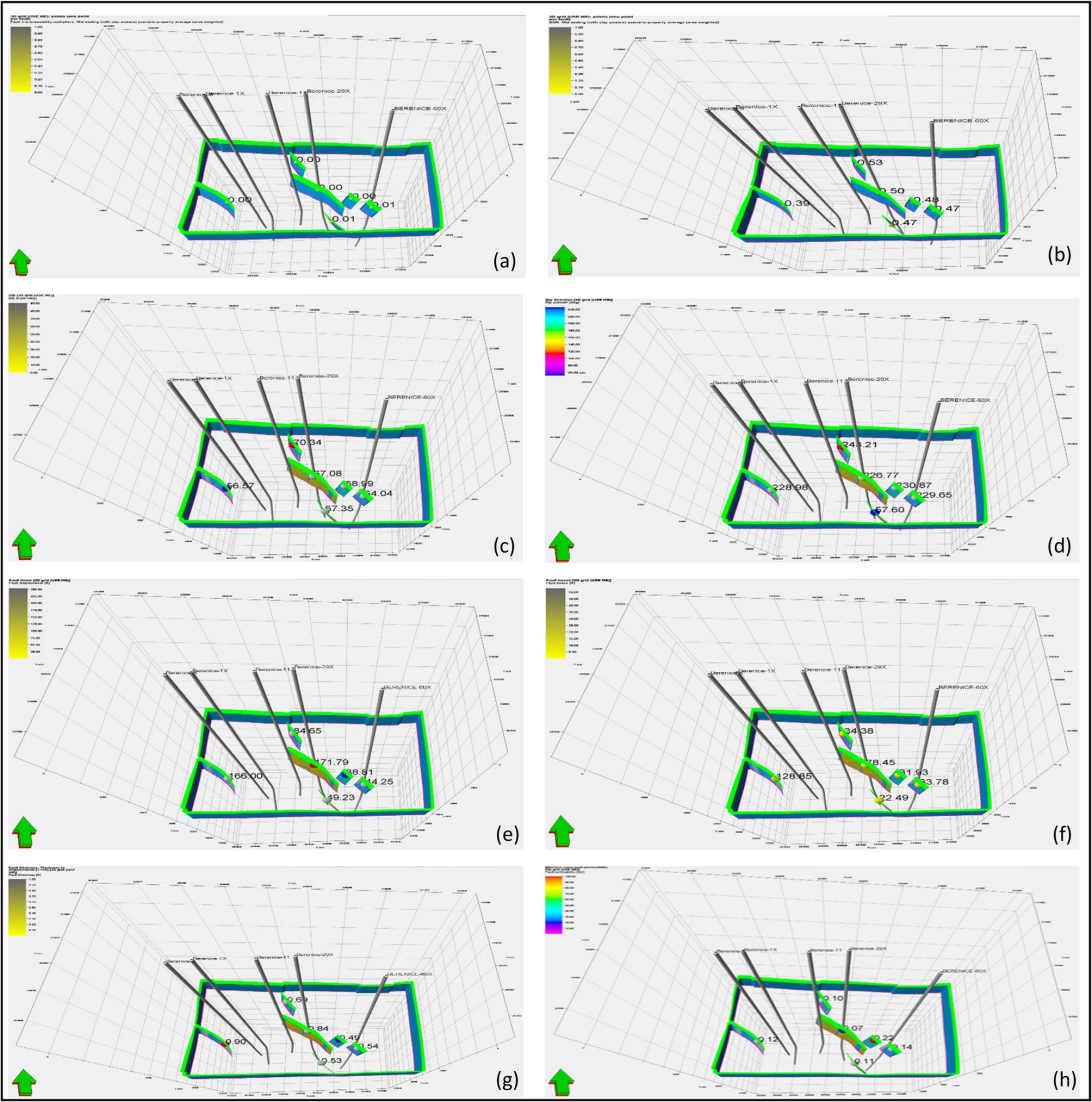
Composite visualization panel of key fault seal attributes and their obtained values: (**a**) fault transmissibility, (**b**) shale gouge ratio, (**c**) fault dip angle, (**d**) fault azimuth, (**e**) fault throw, (**f**) fault heave, (**g**) fault thickness, (**h**) fault permeability.

1. Shale gauge ratio (SGR): at each point on the fault, the net content of shale/clay in the volume of rock that has slipped past that point on the fault is calculated^68^:
  $${\text{SGR }} = \, \Sigma \, \left( {{\text{Vsh }}\left( {\Delta {\mathrm{Z}}/{\mathrm{t}}} \right) \times {1}00} \right)$$
  where; Vsh is the shale volume, Z is the displacement, t is the throw of the fault. It ranges between 0 and 1.
2. Fault zone permeability (Kf): it is the ability of fault to conduct fluids. It can be calculated using the following equation ^69^:
  $${\text{Log K}}_{{\mathrm{f}}} = {\text{4 SGR}} - \left( {{1}/{4}} \right){\mathrm{log}}\left( {\mathrm{D}} \right)\left( {{1} - {\mathrm{SGR}}} \right)$$
  where; K_f_ is fault permeability (in mD) and D is fault displacement (in ft). SGR is shale gauge ratio.
3. Fault transmissibility (T): it is calculated as a function of the dimensions and permeability of the grid blocks and the thickness and permeability of the fault ^69^:
  $$T = \left[ {1 + t_{f} \frac{{\left( {2/k_{f} - 1/k_{i} - 1/k_{j} } \right)}}{{\left( {L_{i} /k_{i} + L_{j} } \right)k_{j} }}} \right]^{ - 1}$$

where $T$ is the Fault transmissibility multiplier, ${t}_{f}$ is Fault tuning (or skin) factor (dimensionless) ${k}_{f}$ is Fault-zone permeability (units: mD or m^2^), ${k}_{i}$ ; ${k}_{j}$ are Permeabilities of the reservoir on one and other Side of the fault, While ${L}_{i}$ ; ${L}_{j}$ Half-distance (or distance) from Point center to the fault plane. Fault transmissibility in the study area ranges from 0 to 1, yet the Bahariya faults exhibit a maximum value of only 0.01 as Shown in (Fig. 17), reflecting exceptionally strong sealing behavior and highly restricted cross-fault flow. This sealing efficiency creates a favorable trapping configuration that enhances hydrocarbon accumulation. Most faults display average SGR values of 0.4–0.5, particularly in the upper sections where hydrocarbon entrapment is most effective. Fault-zone permeability varies between 0.2 and 9 mD, with the majority falling between 0.01 and 0.07 mD, indicating semi-permeable to impermeable fault planes. Structurally, the field is dominated by faults with dip angles of 60°–70° and azimuths between 57° and 243°, defining a predominantly southeastward trend. Fault throws range from 50 to 170 ft, commonly juxtaposing the Bahariya Formation against the shale-rich Abu Roash G Unit. This displacement promotes shale smearing and elevated SGR values, further enhancing fault sealing capacity. Collectively, these characteristics confirm that the faults act as effective barriers and pressure-retaining features, supporting hydrocarbon entrapment within the reservoir system.

Fault seal analysis provides a critical bridge between structural interpretation and reservoir performance, offering a deeper understanding of hydrocarbon distribution within the Bahariya Formation. The NW–SE trending fault system, identified through seismic interpretation and structural mapping, not only defines the geometric framework of the reservoir but also exerts a primary control on fluid migration and trapping efficiency. When integrated with seismic attribute analysis, the spatial coincidence between amplitude anomalies and structurally elevated fault-bounded closures suggests that these faults act as effective lateral seals, enabling hydrocarbon accumulation within discrete compartments.

This interpretation is further supported by petrophysical observations, where zones exhibiting lower water saturation (24 to 25%) and relatively high porosity (26 to 27%) are preferentially located within structurally confined areas. Such distributions indicate restricted fluid communication across fault planes, consistent with sealing behavior. Moreover, the reduction in shale volume toward the western structural closure, as revealed by the 3D geological model, enhances reservoir quality while simultaneously increasing the likelihood of fault-related trapping mechanisms, particularly where shale smearing or clay-rich fault gouge may contribute to seal integrity.

Importantly, the integration of fault seal analysis with net-pay distribution reveals a strong spatial correlation between productive intervals and fault-bounded structural highs, reinforcing the concept that fault architecture governs both reservoir compartmentalization and hydrocarbon retention^70,71^. This multi-scale consistency from seismic-scale structures to petrophysical properties demonstrates that fault sealing capacity is not merely a localized phenomenon but a field-scale controlling factor^72^.

Unlike conventional approaches that treat fault seal analysis as an isolated evaluation, this study demonstrates that incorporating fault sealing behavior within an integrated seismic–petrophysical–modeling workflow significantly enhances the predictive understanding of hydrocarbon occurrence. This approach allows for the identification of high-confidence exploration targets by linking structural geometry, reservoir quality, and fluid distribution within a unified framework. Consequently, fault seal analysis emerges as a key determinant in reducing exploration risk and optimizing development strategies in structurally complex reservoirs such as the Berenice Field.

### Reserves estimation

Reserves estimation in the Berenice Field is a critical step for quantifying recoverable hydrocarbons and assessing the field’s economic viability^73^. This evaluation integrates geological, petrophysical, and volumetric inputs to generate reliable estimates of oil and gas in place. Key parameters net pay, porosity, water and hydrocarbon saturation, and formation volume factors are constrained by well logs, core data, and seismic interpretation^74–76^. Log and test results define the oil–water contact, which occurs at − 5355 ft SSTVD in the Eastern Closure and − 5280 ft SSTVD in the Western Closure. Due to reservoir heterogeneity, the oil column thickness, porosity, and water saturation vary laterally across the field. Modern modeling techniques, supported by high-performance computing, allow rapid and precise reserve calculations. Using an oil FVF of 1.15 and a recovery factor of 0.20, the estimated reserves for the Bahariya sandstone across both three-way dip closures are summarized in Table 1.

Once a field has been discovered, accurate reservoir data become available and a more sophisticated formula may be applied ^77^:

$${\mathrm{Recoverable}}\;{\mathrm{Oil}}\;\left( {{\mathrm{BBL}}} \right) = \frac{{7758v\phi \left( {1 - S_{w} } \right)R}}{FvF}$$

where: V is the volume (area × thickness), 7758 is conversion factor from acre-feet to Barrels, $\phi$ is average effective porosity, SW is average water saturation, R is estimated recovery factor, FVF is formation volume factor.

Bulk volume represents the total rock volume, calculated from the reservoir area and gross thickness. Net volume is derived similarly but uses the net-to-gross thickness. Hydrocarbons in Pore Volume (HCPV) express the volume of hydrocarbons occupying the pore space, obtained by multiplying pore volume by porosity. Stock Tank Oil Initially in Place (STOIIP) is then calculated by multiplying HCPV by hydrocarbon saturation (1 – Sw). Recoverable oil results from applying the recovery factor to STOIIP and represents the portion of hydrocarbons that can be commercially produced. The BAH Formation showed a Fair recoverable oil reserve of 1.7 million stock tank barrel in the Block whereas BERENICE-29X Was Drilled, while Other Block Near BERENICE-1X Showed Notable recoverable oil reserves of 4.07 million stock tank barrel. Also other thin and marginal oil bearing zones in other BAH Blocks are Being assessed Whereas It can add more value to the oil reserve and production on the long run.

Overall, the reserves estimation for the Berenice Field provides a robust basis for production forecasting, development planning, and economic evaluation, ensuring that field development strategies are aligned with the reservoir’s true productive capacity^78^.

### Uncertainty analysis of reserve calculation

Uncertainty is a fundamental challenge in petroleum reserve estimation. When building geological models structural or property-based, several parameters inevitably introduce uncertainty into volumetric calculations^79–84^.

**Fig. 18:**
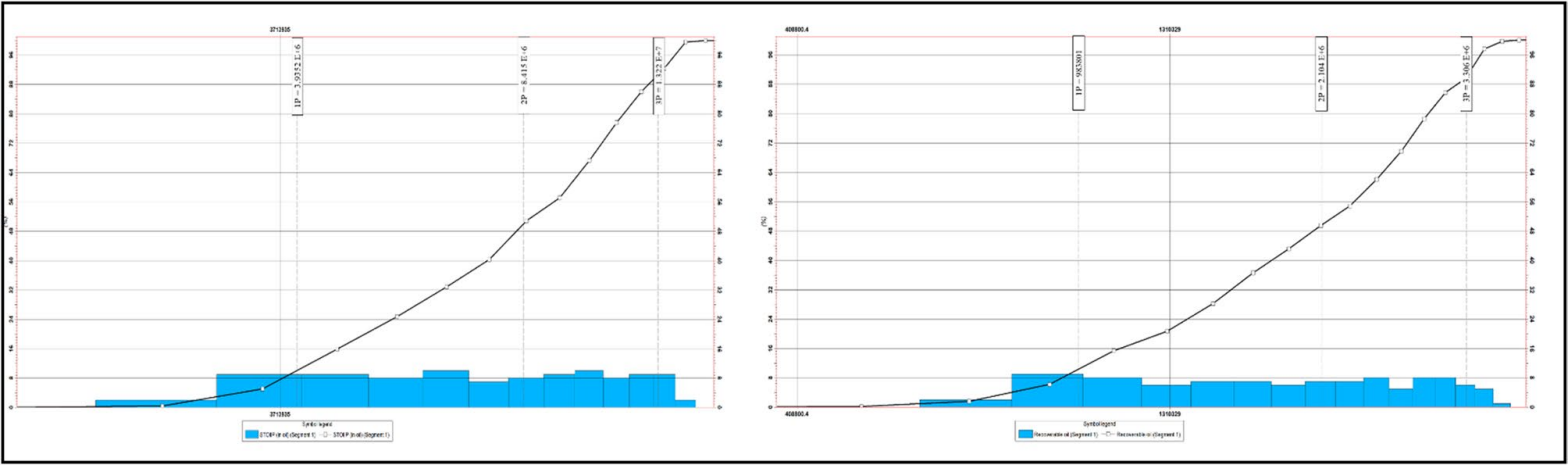
Shows the Histogram and Cumulative Curve For Bahariya Reserves (Eastern Block).

**Fig. 19:**
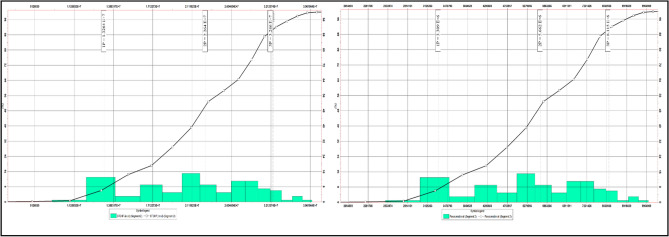
Shows the histogram and cumulative curve for Bahariya reserves (Western Block).

Samimi and Karimi^85^ group these sources into structural interpretation, fluid contacts, petrophysical properties, and key reservoir parameters such as formation volume factors (Bo, Bg). Although formation volume and recovery factors are often treated as constants, reserve estimates remain most sensitive to three variables: Net-to-Gross ratio (N/G), water saturation (Sw), and effective porosity (PHIE). Any geological model contains multiple uncertain inputs; when several are varied simultaneously, numerous realizations must be generated to capture the full range of outcomes^86,87^. For a single variable, only minimum, maximum, and most-likely cases are required. In Petrel, the uncertainty workflow involves defining a base case, running multiple realizations, and evaluating each variable’s impact on volumetrics. Monte Carlo sampling is applied, where random values are drawn within specified ranges, the model is recalculated, and results are stored^85,88–90,91^. Repeating this process here for 100 realizations produce a distribution of outcomes from which probabilistic reserves (P10, P50, P90) can be derived. These probabilities, along with the associated histogram, cumulative curve, and the loop/seed settings for each case, are presented in as Shown in (Figs. 18 and 19) and Tables 2 and 3.

**Table 3:** Shows loop and seed number with recoverable oil in each case.

Item	$Recoverable_oil_10_3_STB_	$SEED	$LOOP
BERENICE_BAH_BLOCK21	5134.6803809909	26,493	43
BERENICE_BAH_BLOCK21	7420.21226590081	6458	44
BERENICE_BAH_BLOCK21	5323.43162140651	14,579	45
BERENICE_BAH_BLOCK21	4743.21303972844	4716	46
BERENICE_BAH_BLOCK21	6154.30461443551	5577	47
BERENICE_BAH_BLOCK21	8457.00109708094	28,490	48
BERENICE_BAH_BLOCK21	7598.1792612063	16,175	49
BERENICE_BAH_BLOCK21	6946.4940717253	25,784	50
BERENICE_BAH_BLOCK21	6691.30335217686	29,988	51

Table 2 shows the recoverable oil arranged in Ascending percentile order, where the value closest to P50 is case 50 with recoverable oil equals 1.6 MMSTB. This value is close to the value of recoverable oil calculated before; which equals (1.77 MMSTB) recoverable oil.

Table 3 shows the recoverable oil arranged in Ascending percentile order, where the value closest to P50 is case 46 with recoverable oil equals 4.473 MMSTB. This value is close to the value of recoverable oil calculated before; which equals (4.07 MMSTB) recoverable oil.

Reserves estimation in this study extends beyond conventional volumetric calculations by incorporating a fully integrated framework of Different Tools to constrain hydrocarbon volumes with higher confidence. The volumetric assessment, traditionally dependent on static parameters such as porosity, water saturation, and net pay, is here dynamically informed by the spatial distribution of these properties within the 3D geological model, resulting in a more realistic representation of reservoir behavior.

Furthermore, the incorporation of fault seal analysis introduces a critical constraint on volumetric calculations by defining the effective boundaries of hydrocarbon accumulation. Faults with higher sealing capacity limit fluid leakage and preserve hydrocarbon columns, thereby increasing the effective reserves within compartmentalized zones. This integration highlights that reserves are not solely controlled by reservoir quality but are equally dependent on structural integrity and sealing efficiency.

The application of uncertainty analysis further enhances the robustness of the reserves estimation by quantifying the variability associated with key input parameters, including porosity, saturation, and structural depth. This probabilistic perspective allows for a more realistic assessment of recoverable volumes and reduces the risk of overestimation or underestimation.

Unlike traditional approaches that treat reserves estimation as a terminal step, this study demonstrates that it can serve as a diagnostic tool for validating the entire reservoir characterization workflow. By linking volumetric outputs directly to seismic, petrophysical, and structural inputs, the approach provides a holistic understanding of hydrocarbon potential and enables the identification of high-confidence exploration targets. Consequently, reserves estimation emerges not only as a measure of hydrocarbon volume but as a critical integrator of multidisciplinary data, significantly improving decision-making in exploration and field development.

### Leads and prospects

**Fig. 20:**
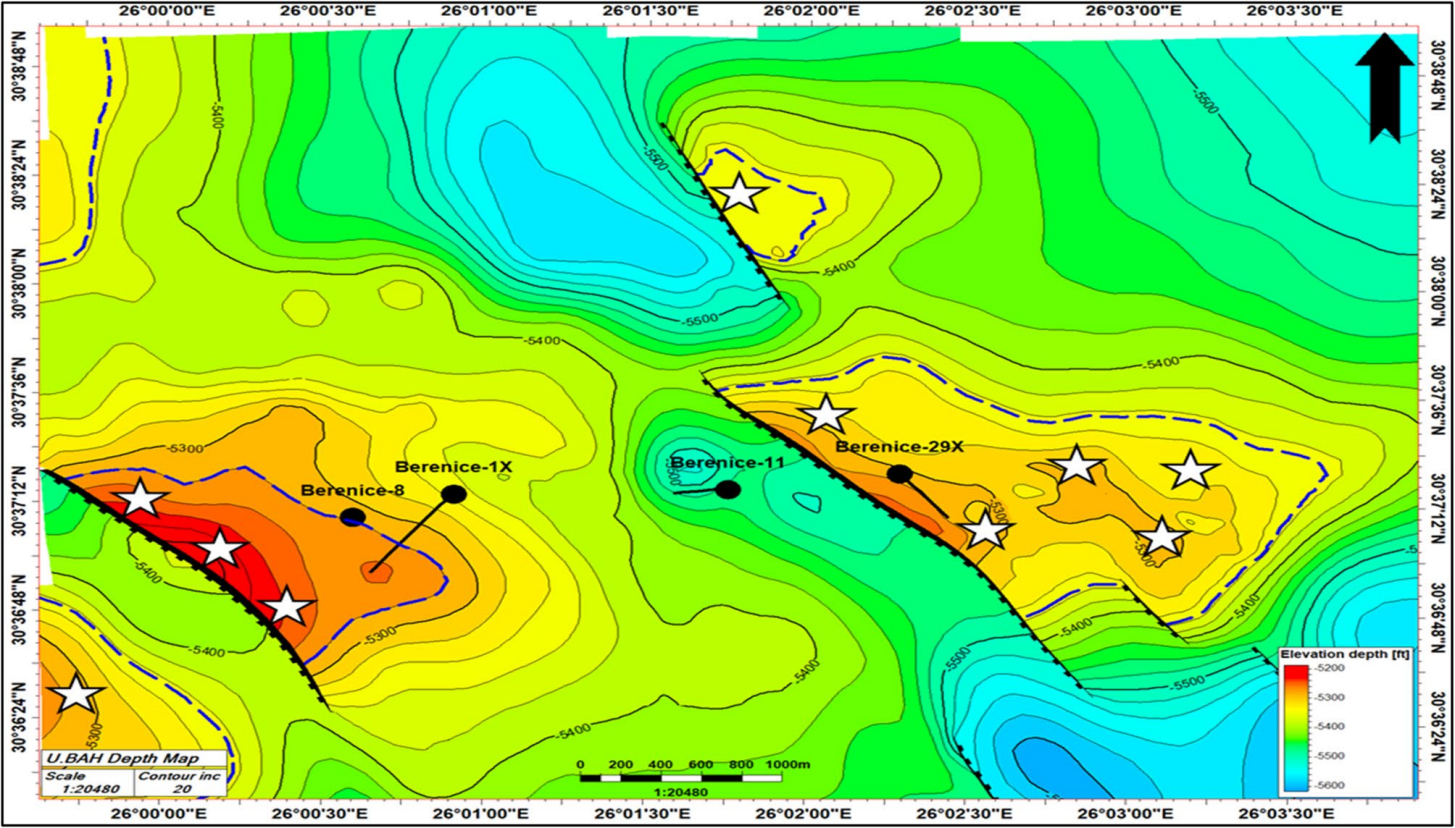
Shows the recommended leads and prospects based on this study.

Integrated seismic, petrophysical, and fault-seal analyses highlight structurally elevated closures within the Bahariya Formation as prime exploration targets. These zones within the Bahariya Formation indicated with (white stars on the depth-structure map) (Fig. 20). These leads are situated in structurally elevated closures bounded by NW–SE normal faults exhibiting effective sealing capacity, providing robust hydrocarbon traps. They correspond with zones of enhanced reservoir quality, characterized by elevated effective porosity (26–27%), low water saturation (~ 24–25%), and reduced shale content, optimizing storage and flow potential. Seismic attributes and amplitude anomalies corroborate the likelihood of hydrocarbon accumulations. This approach quantifies the interplay between reservoir heterogeneity and fault-controlled compartmentalization, highlighting areas where structural integrity, facies continuity, and migration pathways converge. Collectively, these leads represent strategically prioritized targets for future appraisal drilling and field development.

### Implications for future exploration

These uncertainties impact hydrocarbon exploration and production. Incorrect estimation of fault geometry or trap definition may result in drilling dry wells or failing to predict the correct recoverable volumes.

## Conclusion

*Structural control of hydrocarbons*: Seismic interpretation and attribute analysis revealed that NW–SE trending normal faults dominate the structural trapping mechanisms, controlling hydrocarbon accumulation in the Berenice Field.

*Reservoir quality and heterogeneity*: Petrophysical and facies modeling demonstrated significant lateral and vertical variability, with porosity up to 26%, net pay up to 10 ft, and low water saturation zones highlighting sweet spots for production.

*Fault seal impact*: Fault seal analysis confirmed that variations in fault geometry, throw, and shale content significantly influence trap integrity and compartmentalization, emphasizing the critical role of structural evaluation in reservoir management.

*Integrated volumetric assessment*: Combined reserves estimation and uncertainty analysis provided reliable predictions of recoverable hydrocarbon volumes, offering actionable insights for field development and exploration planning.

*Methodological contribution*: The study establishes a comprehensive workflow integrating seismic, petrophysical, and modeling techniques, providing a template for future evaluation of structurally complex clastic reservoirs.

Overall, the multidisciplinary approach adopted in this study underscores the critical role of integrated seismic, petrophysical, and modeling techniques in achieving a robust reservoir characterization of the Berenice Field. The results not only improve the understanding of the field’s structural and stratigraphic complexity but also offer valuable insights for future exploration, development planning, and production optimization within the Western Desert region.

## Data Availability

The datasets underpinning this study were supplied by the Egyptian General Petroleum Corporation. Owing to licensing constraints, they are not available for public release. Access may be granted upon reasonable request to the corresponding author, subject to prior authorization from the Egyptian General Petroleum Corporation.

## References

[1] Said, R. *The Geology of Egypt* (A.A. Balkema, 1990).

[2] Egyptian General Petroleum Corporation (EGPC). Western desert, oil and gas fields. A comprehensive overview. Presented at 11th EGPC petrol. Explor. Prod. Conf. (1992).

[3] Meshref, W. M. Tectonic framework of Egypt. In *Geology of Egypt* (ed. Said, R.) 113–156 (Balkema, 1990).

[4] Acharyya, S. K. & Roy, A. Tectonothermal history of the Central Indian Tectonic Zone and reactivation of major faults/shear zones. *J. Geol. Soc. India* **55**, 239–256 (2000).

[5] Nour eldin, A. M., Mabrouk, W. M. & Metwally, A. M. Superimposed structure in the southern periphery of Abu Gharadig Basin, Egypt: Implication to petroleum system. *Contrib. Geophys. Geodesy.* **53**(2), 97–110 (2023).

[6] El Awdan, A., Youssef, F. & Moustafa, A. R. Effect of mesozoic and tertiary deformations on hydrocarbon exploration in the Northern Western desert, Egypt. In *Am. Assoc. Pet. Geol Int. Meet.* (2002).

[7] Abd-Allah, Z. M., Maky, A. F. & Ramadan, M. A. Organic source of crude oils and 1D basin modeling of upper Cretaceous rocks, Badr Concession, Abu Gharadig Basin, Western Desert, Egypt. *Arab. J. Geosci.* **11**, 1–13 (2018).

[8] Moustafa, A. R. Mesozoic-Cenozoic basin evolution in the Northern Western Desert of Egypt. *Geol. East. Libya* **3**, 29–46 (2008).

[9] Cheng, J. E. Petroleum system of Shoushan Basin, Western Desert, Egypt. *Acta Sci. Malay.* **4**(1), 1–8 (2020).

[10] Sultan, N. & Halim, M. A. Tectonic framework of northern Western Desert, Egypt and its effect on hydrocarbon accumulations. *EGPC Bull.* **2**, 1–19 (1988).

[11] Schlumberger. *Log Interpretation Principles, Applications* (Schlumberger Wireline & Testing, 1984).

[12] Schlumberger. *Log Interpretation Charts* (Schlumberger Wireline & Testing, 1995).

[13] World Energy Conference (WEC). *Survey of Energy Resources* (World Energy Conference, 1984).

[14] Meshref, W. M. & El Sheikh, A. A. *Tectonic Framework of Egypt and its Interpretation* (Egyptian General Petroleum Corporation (EGPC), 1973).

[15] Aal, A. A. et al. Tectonic evolution of the eastern Mediterranean Basin and its significance for hydrocarbon prospectivity. *GeoArabia* **5**, 363–384 (2000).

[16] Tari, G., Shahin, A. & Baur, J. Mid-Cretaceous rifting in the Levant Basin. *Mar. Pet. Geol.* **28**, 726–742 (2012).

[17] Acharyya, S. K. The nature of Mesoproterozoic Central Indian Tectonic Zone with exhumed and reworked older granulites. *Gondwana Res.* **6**(2), 197–214 (2003).

[18] Bian, Q., Zhang, J. & Huang, C. The middle and lower Cambrian salt tectonics in the central Tarim Basin, China: A case study based on strike-slip fault characterization. *Energy Geosci.* **5**(2), 100254 (2024).

[19] Turner, R. et al. Structural and stratigraphic controls on reservoir architecture: A case study from the lower Oligocene Vicksburg Formation, Brooks County, Texas. *Mar. Pet. Geol.* **160**, 106627 (2024).

[20] Soliman, M. & El Badry, O. *Geology of the Nile Delta* (Egyptian General Petroleum Corporation (EGPC), 1980).

[21] El Gezeery, M. N. & O’Connor, D. *Geology of the Gulf of Suez Region* (Egyptian General Petroleum Corporation (EGPC), 1975).

[22] Bosworth, W., Huchon, P. & McClay, K. The Red Sea and Gulf of Aden basins. *J. Afr. Earth Sci.* **43**, 334–378 (2015).

[23] Ali, M., Zhu, P., Jiang, R., Huolin, M. & Ashraf, U. Improved prediction of thin reservoirs in complex structural regions using post-stack seismic waveform inversion: A case study in the Junggar Basin. *Can. Geotech. J.* **61**, 2839 (2024).

[24] Alcalde, J. et al. The importance of structural model availability on seismic interpretation. *J. Struct. Geol.* **97**, 161–171 (2017).

[25] Abu deif, A. M., Attia, M. M., Al-Khashab, H. M. & Radwan, A. E. Hydrocarbon type detection using the synthetic logs: A case study, Baba Member, Gulf of Suez, Egypt. *J. Afr. Earth Sci.* **144**, 176–182 (2018).

[26] Mustafa, A., Alfarraj, M., AlRegib, G. Estimation of Acoustic Impedance from Seismic Data using Temporal Convolutional Network. In *Expanded Abstracts of the SEG Annual Meeting* (San Antonio, TX, 2019).

[27] Totake, Y., Butler, R. W. & Bond, C. E. Structural validation as an input into seismic depth conversion to decrease assigned structural uncertainty. *J. Struct. Geol.* **95**, 32–47 (2017).

[28] Noureldin, A. M., Mabrouk, W. M. & Metwally, A. Delineating tidal channel feature using integrated post-stack seismic inversion and spectral decomposition applications of the Upper Cretaceous reservoir Abu Roash C: A case study from Abu-Sennan Oil Field, Western Desert, Egypt. *J. Afr. Earth Sci.* **205**, 104974 (2023).

[29] La Marca, K., Bedle, H., Stright, L. & Marfurt, K. Sensitivity analysis of seismic attributes parametrization to reduce misinterpretations: Applications to deepwater channel complexes. *Mar. Pet. Geol.* **153**, 106309 (2023).

[30] Fashagba, I., Enikanselu, P., Lanisa, A. & Matthew, O. Seismic reflection pattern and attribute analysis as a tool for defining reservoir architecture in ‘SABALO’ Field, deepwater Niger Delta. *J. Pet. Explor. Prod. Technol.* **10**, 991–1008 (2020).

[31] El-Sayed, A. S., Mabrouk, W. M. & Metwally, A. M. Utilizing post-stack seismic inversion for delineation of gas-bearing sand in a Pleistocene reservoir, Baltim gas field, Nile Delta, Egypt. *Sci. Rep.* **14**(1), 29596 (2024). 39609520 10.1038/s41598-024-78186-9 PMC11605053

[32] Abuzaied, M., Metwally, A., Mabrouk, W., Khalil, M. & Bakr, A. Seismic interpretation for the Jurassic/Paleozoic reservoirs of QASR gas field, Shushan-Matruh Basin North Western Desert, Egypt. *Egypt. J. Pet.* **28**(1), 103–110 (2019).

[33] Hussain, S. et al. A comprehensive study on optimizing reservoir potential: Advanced geophysical log analysis of Zamzama Gas Field, southern Indus Basin, Pakistan. *Phys. Chem. Earth A/B/C* **135**, 103640 (2024).

[34] Metwalli, F. I., Shendi, E. A. H. & Fagelnour, M. S. Seismic facies analysis of thin sandstone reservoirs, North Western Desert, Egypt. *J. Pet. Explor. Prod. Technol.* **9**, 793–808 (2019).

[35] Faleide, T. S. et al. Impacts of seismic resolution on fault interpretation: Insights from seismic modelling. *Tectonophysics* **816**, 229008 (2021).

[36] Gul, M. A., Awan, R. S., Khan, A., Iltaf, K. H. & Butt, S. H. E. 2D seismic interpretation of Sawan Gas Field integrated with petrophysical analysis: A case study from Lower Indus Basin. Pakistan. *Energy Geoscience* **4**, 100143 (2023).

[37] Al-Chalabi, M. Time-depth relationships for multilayer depth conversion. *Geophys. Prospect.* **45**, 715–720 (1997).

[38] Asquith, G. B. & Gibson, C. R. *Basic Well Log Analysis for Geologists: American Association of Petroleum Geologists Tulsa* 216. (1982).

[39] Hassan, M., Mabrouk, W. M. & Farhoud, M. Petrophysical analysis for Ammonite-1 well, Farafra area, Western Desert, Egypt. *Arab. J. Geosci.* **7**(12), 5107–5125 (2014).

[40] Sadeq, Q. M. & Yusoff, W. I. W. B. W. Carbonate reservoirs petrophysical analysis of Bai Hassan oil field North of Iraq. *J. Bioremediat. Biodegrad.* **6**, 1 (2015).

[41] Stephens, D. B. et al. A comparison of estimated and calculated effective porosity. *Hydrogeol. J.* **6**, 156–165 (1998).

[42] Ehrenberg, S. N., Eberli, G. P., Keramati, M. & Moallemi, S. A. Porosity-permeability relationships in interlayered limestone-dolostone reservoirs. *AAPG Bull.* **90**, 91–114 (2006).

[43] Li, H. et al. Quantitative characterization of complex multi-scale fractures in low-permeable sandstone reservoir: Insights from geological and mathematical approach. *Geomech. Geophys. Geo-Energy Geo-Resour* **12**, 39 (2026).

[44] Metwally, A. M., Mabrouk, M. & Mahmoud, I. A numerical approach to accurately estimate water resistivity (Rw) and saturation (Sw) in shaly sand formations. *Contrib. Geophys. Geodesy.* **52**(3), 423–441 (2022).

[45] Chikiban, B., Kamel, M. H., Mabrouk, M. & Metwally, A. M. Petrophysical characterization and formation evaluation of sandstone reservoir: Case study from Shahd field, Western Desert, Egypt. *Contrib. Geophys. Geodesy.* **52**(03), 443–466 (2022).

[46] Sarhan, M. A. & Abdel-Fattah, M. I. Integrating well logs and seismic data for a comprehensive geophysical appraisal of post Albian oil reservoirs in the SWQ-4X well, Gindi Basin, Egypt. *Egypt. J. Pet.* **33**(2), 2 (2024).

[47] Sarhan, M. A. & Abdel-Fattah, M. I. Geophysical evaluation and petrophysical assessment of the Abu Roash F Member: A probable unconventional oil reservoir in Heba Field, Eastern Abu Gharadig Basin, Egypt. *J. Afr. Earth Sci.* **217**, 105330 (2024).

[48] Metwally, M. et al. Formation evaluation of Abu Madi reservoir in Baltim Gas Field, Nile Delta, using well logs, core analysis, and pressure data. *Sci. Rep.* **13**(1), 19139 (2023). 37932367 10.1038/s41598-023-46039-6 PMC10628261

[49] Gobia, H. B. & Alazrag, A. A Heterogeneous carbonate characterization using integration of petrophysical analysis to different rock type approaches, a case study in the carbonate of upper Sabil and Harash formations, Intisar-D Field, Concession 103 Sirte Basin, Libya. In *Soc Pet Eng—Mediterr Offshore Conf MOCE* (2024).

[50] Anyiam, O. A., Mode, A. W. & Okara, E. S. The use of cross-plots in lithology delineation and petrophysical evaluation of some wells in the Western Coastal Swamp, Niger Delta. *J. Pet. Explor. Prod. Technol.* **8**, 61–71 (2018).

[51] Lasheen, I., Noureldin, A. M. & Metwally, A. Reservoir characterization of the Abu Roash D Member through petrography and seismic interpretations in Southern Abu Gharadig Basin, Northern Western Desert, Egypt. *Sci. Rep.* **14**(1), 8966 (2024). 38637582 10.1038/s41598-024-58846-6 PMC11026448

[52] Al-Qayim, B. & Rashid, F. Reservoir characteristics of the Albian Upper Qamchuqa Formation carbonates, Taq Taq oilfield, Kurdistan, Iraq. *J. Pet. Geol.* **35**, 317–341 (2012).

[53] Calcagno, P., Chilès, J. P., Courrioux, G. & Guillen, A. Geological modelling from field data and geological knowledge Part I. Modelling method coupling 3D potential-field interpolation and geological rules. *Phys. Earth Planet. Inter.* **171**(1–4), 147–157 (2008).

[54] Mahdi, M. M., Ismail, M. J. & Mohammad, O. A. M. The integration of wireline logs and sedimentological data to predict sequence stratigraphic framework in carbonate rocks: An example from Rumaila Formation (Cenomanian), West Qurna oil Field, Southern Iraq. *Stratigr. Geol. Correl.* **30**, 360–377 (2022).

[55] Abdel-Fattah, M. & Tawfik, A. 3D geometric modeling of the Abu Madi reservoirs and its implication on the gas development in Baltim area (Ofshore Nile Delta, Egypt). *Int. J. Geophys.* **11**, 369143 (2015).

[56] Okoli, E. A., Ukaegbu, V. U. & Omoboriowo, A. O. Evaluation of petrophysical properties and hydrocarbon reservoir potential using well log data in the Niger Delta. *Niger. J. Sci. Environ.* **19**(2), 96–105 (2021).

[57] Fallah-Bagtash, R. et al. Integrated petrophysical and microfacies analyses for a reservoir quality assessment of the *Asmari dolostone* sequence in the Khesht Field, SW Iran. *J. Asian Earth Sci.* **223**, 104989 (2022).

[58] Abdel-Fattah, M. I., Metwalli, F. I. & El Sayed, I. M. Static reservoir modeling of the Bahariya reservoirs for the oilfields development in South Umbarka Area, Western Desert, Egypt. *J. Afr. Earth Sci.* **138**, 1–13 (2018).

[59] Fagelnour, M. S., Metwalli, F. I. & Shendi, E. A. H. Structural and facies modeling of the Lower Cretaceous Alam El Bueib Reservoirs in the Shushan Basin, Western Desert, Egypt. *Arab. J. Geosci.* **11**(18), 553 (2018).

[60] Chen, Q. Y., Liu, G., Ma, X. G., Li, X. C. & He, Z. W. 3D stochastic modeling framework for Quaternary sediments using multiple-point statistics: A case study in Minjiang Estuary area Southeast China. *Comput. Geosci.* **136**, 104404 (2020).

[61] Abuzaied, M., Mabrouk, W. M., Metwally, A. M., Bakr, A. & Eldin, S. E. Correlation of the reservoir characteristics from the well logging data and core measurements in QASR field, North Western Desert Egypt. *Arab. J. Geosci.* **13**, 1–9 (2020).

[62] Lindsay, M., Ailleres, L., Jessell, M. W., de Kemp, E. & Betts, P. G. Locating and quantifying geological uncertainty in three-dimensional models: Analysis of the Gippsland Basin, southeastern Australia. *Tectonophysics* **546–547**, 10–27 (2012).

[63] Molenaar, M. M. et al. Applying Geo-Mechanics and 4D: 4D In-Situ Stress as a Complementary Tool for Optimizing Field Management. In *ARMA North America Rock Mechanics Symposium ARMA *(2004).

[64] Freeman, B. et al. Fault seal prediction: The gouge ratio method. *Geol. Soc. Lond. Spec. Publ.* **127**(1), 19–25 (1998).

[65] Harris, D. et al. Using shale gouge ratio (SGR) to model faults as transmissibility barriers in reservoirs: An example from the Strathspey Field, North Sea. *Pet. Geosci.* **8**(2), 167–176 (2002).

[66] Bretan, P., Yielding, G. & Jones, H. Using calibrated shale gouge ratio to estimate hydrocarbon column heights. *AAPG Bull.* **87**(3), 397–413 (2003).

[67] Indrarto Y. B. C. *Fault-Seal Analysis in Minas Field Area-1, Central Sumatra Basin*. (2014).

[68] Yielding, G., Freeman, B. & Needham, D. T. Quantitative fault seal prediction. *AAPG Bull.* **81**(6), 897–917 (1997).

[69] Manzocchi, T. The connectivity of two-dimensional networks of spatially correlated fractures. *Water Resour. Res.* **35**, 193–199 (1999).

[70] Sapiie, B. et al. Problems in conducting fault seal analysis in carbonate reservoir. In *Proceedings, Indonesian Petroleum Association, Forty-First Annual Convention & Exhibition* (2017).

[71] Walker, C. D. & Evenick, J. C. Understanding reservoir compartmentalization using shale gouge ratio. *Dev. Struct. Geol. Tecton.* **5**, 225–230 (2019).

[72] Fan, X. et al. Application of Shale Gouge ratio method in prediction of fault sealing property in block A. In *International Field Exploration and Development Conference* (Singapore, Springer Nature Singapore, 2021).

[73] Cronquist, C. *Estimation and Classification of Reserves of Crude Oil, Natural Gas, and Condensate* (2001).

[74] Barakat, M. K. & Nooh, A. Z. Reservoir quality using the routine core analysis data of Abu Roash C in Badr El Din-15 oil field, Abu Gharadig basin, North Western Desert, Egypt. *J. Afr. Earth Sci.* **129**, 683–691 (2017).

[75] Metwalli, F. I., Shendi, E. A. H. & Fagelnour, M. S. Core and well logs interpretation for better reservoir characterization in Shushan Basin, Egypt. *Arab. J. Geosci.* **14**, 1–14 (2021).

[76] Osman, W., Kassab, M. A., Elgibaly, A. A. & Samir, H. Petrophysical evaluation of sandstone gas reservoir using integrated well logs and core data for the lower Cretaceous Kharita Formation, Western Desert, Egypt. *J. Pet. Explor. Prod. Technol.* **11**(3), 969–982 (2021).

[77] Selley, R. C. *Elements of Petroleum Geology* (Academic Press, 1998).

[78] Abdel-Fattah, M., Hanafy, M., Hamdan, H. & Attia, T. Integrated three dimensional reservoir modeling and tectonic evaluation of the Upper Cretaceous Bahariya Formation in the Burg El Arab area, Egypt: An implications for hydrocarbon exploration and production strategies. *Egypt. J. Pet.* **33**(2), 259–268 (2024).

[79] Li, H. et al. Progressive geological modeling and uncertainty analysis using machine learning. *ISPRS Int. J. Geo-Inf.* **12**(3), 97 (2023).

[80] Amer, M., Mabrouk, W. M., Soliman, S., Noureldin, M. & Metwally, A. Tree-dimensional integrated geo-static modeling for prospect identification and reserve estimation in the Middle Miocene multi-reservoirs: A case study from Amal Field, Southern Gulf of Suez Province. *Nat. Resour. Res.* **32**(6), 2609–2635 (2023).

[81] Korotaev, M. V., Pravikova, N. V. & Aleshina, K. F. 3D modeling of sedimentation in clinoform complexes of the North Chukchi Basin. *Moscow Univ. Geol. Bull.* **80**(5), 89–95 (2024).

[82] Satter, A. & Iqbal, G. M. Reservoir fluid properties. In *Reservoir Engineering* 81–105 (2016).

[83] Yang, Y. et al. A case study of subsurface uncertainty analysis in modelling carbonate reservoir. In *Proceedings of the International Field Exploration and Development. SSGG* (ed. Lin, J.) 851–864 (Springer, Singapore, 2020).

[84] Clarkson, C. R. *Unconventional Reservoir Rate-Transient Analyis* (Gulf Professional Publishing, 2021).

[85] Samimi, A. K. & Karimi, G. Sensitivity & uncertainty analysis of original oil-in-place in carbonate reservoir modelling, a case study. *Pet. Coal* **56**(3), 332–338 (2014).

[86] Sarhan, M. A., Shehata, A. A. & Abdel-Fattah, M. I. Sequence stratigraphic and petrophysical controls on the oil-reservoirs architecture: A case study from the Cretaceous meqasequence, Gulf of Suez region, Egypt. *J. Afr. Earth Sci.* **219**, 105412 (2024).

[87] Sedki, A., Abdelhady, M. A. E. M., Ahmed, H. E. & Reda, M. Petrophysical evaluation and 3D reservoir modeling in Qawasim formation, El Basant gas field, Nile Delta, Egypt: Insights from well-log analysis and 2D seismic data. *Arab. J. Geosci.* **18**, 54 (2025).

[88] Bueno, J. F. et al. Constraining uncertainty in volumetric estimation: A case study from Namorado field, Brazil. *J. Pet. Sci. Eng.* **77**(2), 200–208 (2011).

[89] Leahy, G. M. & Skorstad, A. Uncertainty in subsurface interpretation: A new workflow. *First Break* **31**(9), 87–93 (2013).

[90] Adeloye, M. O. et al. Quantifying uncertainty in oil reserves estimate. *Res. J. Eng. Sci.* **4**(12), 1–8 (2015).

[91] Amer, M., Mabrouk, W. M., Eid, A. M. & Metwally, A. Petrophysical assessment of the Hammam Faraun, Matulla and Nubia reservoirs in the Ashrafi oil field, Gulf of Suez. *Sci. Rep.* **15**(1), 3326 (2025). 39865084 10.1038/s41598-025-86297-0 PMC11770147

